# Appropriate empiric antibiotic choices in health care associated urinary tract infections in urology departments in Europe from 2006 to 2015: A Bayesian analytical approach applied in a surveillance study

**DOI:** 10.1371/journal.pone.0214710

**Published:** 2019-04-25

**Authors:** Zafer Tandogdu, Evgenios T. A. Kakariadis, Kurt Naber, Florian Wagenlehner, Truls Erik Bjerklund Johansen

**Affiliations:** 1 Institute for Clinical Medicine, Oslo University, Oslo, Norway; 2 Northern Institute for Cancer Research, Newcastle University, Newcastle-upon-Tyne, United Kingdom; 3 School of Mathematics, Newcastle University, Newcastle-upon-Tyne, United Kingdom; 4 Munich Technical University, Munich, Germany; 5 Department of Urology, Pediatric Urology and Andrology, Justus Liebig University, Giessen, Germany; 6 Department of Urology, Oslo University Hospital, Oslo, Norway; University Medical Center Utrecht, NETHERLANDS

## Abstract

**Background:**

Health care associated urinary tract infections (HAUTI) is a common complicating factor of urological practice. It is unclear what the appropriate empirical antibiotic choices are and how infection control policies (ICP) influence this. The aim of this study is to use probabilistic approaches towards the problem. That is to determine the chances of coverage of empirical antibiotic choices in HAUTIs and their annual trends in Europe. In addition, the impact of departmental self-reported compliance with catheter management and regulated usage of prophylactic antibiotics policies was tested. The estimated chances of coverage of antibiotics and further probabilistic calculations are carried out using the Global Prevalence of Infections in Urology (GPIU) annual surveillance study European data.

**Methods:**

GPIU is a multi-state annual prevalence study conducted in urology departments to detect patients with HAUTIs, using the Center for Disease Control (CDC) definitions and antimicrobial resistance (AMR). In this analysis; the European cohort from 2005 to 2015 was used. The estimated chance of coverage for each antibiotic choice in HAUTIs was calculated using the Bayesian Weighted Incidence Syndromic Antibiogram (WISCA) approach. Annual trend of the overall cohort and number of appropriate antibiotic choices were estimated. Departments were compared according to their self-reported compliance to ICPs to determine if there was an impact on chances of coverage and appropriate antibiotic choices.

**Results:**

We estimated that in most study years less than half of the single agent antibiotics and all combination options were appropriate for empirical treatment of HAUTIs. Departments with compliance to both ICPs were estimated to have 66%(2006) to 44% (2015) more antibiotic choices compared to departments with complete lack of compliance to the ICPs. In our estimates departments with adherence to a single policy was not superior to departments with complete lack of adherence to ICPs.

**Conclusions:**

Most single agent choices had limited coverage for HAUTIs and combination choices had improved chance of coverage. Optimal antibiotic selection decision should be part of decision experiments and tested in local surveillance studies. Departments with self-reported compliance to ICPs have more antibiotic choices and details of the compliance should be evaluated in future studies. The analysis herein showed that over the 10-year course there was no clear time trend in the chances of coverage of antibiotics (Bayesian WISCA) in European urology departments.

## Introduction

Health care associated urinary tract infections (HAUTI) in urology are a complicating factor of health care and their prevalence is estimated to be 7.7% [1]. Unfortunately, their prevention by using prophylaxis and treatment with antibiotics is hindered by the high levels of antimicrobial resistance (AMR). Despite recommendations on prudent use of antibiotics, misuse of antibiotics is an ongoing issue and escalates AMR in health care [2, 3]. Tackling AMR is of paramount importance to maintain availability of efficacious antibiotics. One of the recommended strategies to tackle AMR is to conduct surveillance of AMR to help selection of appropriate empirical antibiotic treatment and improve policy making [4].

Although, surveillance data is useful for clinical decision making (stewardship) and policy development, has some limitations. The main one is derived from the conventional approaches to synthesize AMR data, which focus on the pathogens and their susceptibility profile for each antibiotic choice. Whereas, from a stewardship and clinical point of view for selecting the appropriate empirical treatment the more relevant question is: “Which antibiotic works best for the condition being treated?”. An alternative way to answer this question is through a compound measure called weighted incidence syndromic combination antibiograms (WISCA) [5]. This is derived from surveillance data and calculated by obtaining the cumulative sum of the relative incidence of each pathogen multiplied with the chances of susceptibility. The WISCA tool provides the expected coverage of a select antibiotic regimen in terms of probabilities and allows direct comparison between different antibiotics on the same scale.

While, WISCA can improve utility of AMR surveillance information it cannot adequately handle information regarding rare pathogens and sequential surveillance data. Within a finite sample obtained from surveillance, rare pathogens might not be present although the likelihood of them causing an infection would still exist. Therefore, prior information regarding these rare instances becomes highly relevant and the *Bayesian* analytical approach can adequately address this issue. In brief, the *Bayesian* approach treats each time point as a set of prior information to build up towards the current information [6]. Therefore, integrating the *Bayesian* approach within the WISCA framework can facilitate that rare pathogens are accounted for [5]. The approach has previously been used to select appropriate empirical antibiotics for pediatric population with blood stream infections [5].

As part of tackling AMR, it’s also important to prevent misuse of antibiotics by improving appropriate antibiotic selection. As such it is useful for policy makers and stewardship programs to provide recommendations of appropriate antibiotic choices for certain conditions. An appropriate antibiotic choice should provide coverage to the causative pathogens. The *Bayesian* WISCA approach can determine the antibiotics choices that are more likely to be appropriate for empirical treatment. This can be achieved by selecting antibiotics with *Bayesian* WISCA values closer to 1 (absolutely appropriate), while an antibiotic with a *Bayesian* WISCA closer to or equal to 0 (absolutely inappropriate) harbors a high chance of failure.

As part of an international collaborative working group (Global Prevalence of Infections in Urology–GPIU) for HAUTIs we have extensively reported on AMR [7–10], albeit accompanied with the aforementioned limitations. HAUTIs are considered as complicated urinary tract infections (UTI) and are associated with an increased morbidity and mortality rates [11]. The GPIU study is an annual surveillance study of HAUTIs in urology departments repeated every year. A key feature of this study is the validated definitions used to capture episodes of HAUTIs. In our previous analysis we illustrated high rates of AMR for both single antibiotics and combination choices [7]. Although, these findings were useful we have been looking for a better way to utilize the data and decided to explore the *Bayesian* WISCA approach, which will allow identifying the appropriate antibiotics and thereby improve empirical treatment of HAUTIs.

While appropriate use of antibiotics is crucial for efficacious treatment of patients with HAUTIs, implementation of infection control policies (ICP) are of utmost importance to prevent AMR and preserve effective antibiotics for the society. In departments with low compliance to ICPs the AMR rates are expected to be higher compared to departments with better compliance. Thus, the chances of coverage of empirical choices would be expected to decline as the level of compliance with ICPs drop [12]. Unfortunately, measurement of the benefits of ICPs is challenging and costly but here, the *Bayesian* WISCA approach could be an alternative to compare different policies. We hypothesized that the self-reported ICP compliance would impact on the *Bayesian* WISCA estimates and available choices of appropriate antibiotics.

In this study the primary aim was to determine the annual *Bayesian* WISCA of empirical antibiotic choices for HAUTIs in European urology departments and to utilize the novel analytic approach to estimate the appropriate antibiotic choices. The secondary aim was to use this probabilistic method to determine the impact of self-reported adherence to ICPs on the available appropriate empirical antibiotic choices in HAUTIs. We used the GPIU study sequential surveillance European data from 2005 to 2015 to estimate the Bayesian WISCA [1, 7–10, 13].

## Material and methods

Data was obtained from an annual global surveillance study of HAUTIs conducted in urology departments (GPIU study). For the current study data obtained from European centers only (2005 to 2015) were used [7, 13]. The three objectives of this study were to;

- Estimate the *Bayesian* WISCA of antibiotic choices and their time trend in HAUTIs;
- Estimate the appropriate antibiotic choices for HAUTIs; and
- Estimate the impact of the reported ICPs adherence on chances of coverage of antibiotic choices.

For the antibiotic choices the three outcomes that overlap with the objectives are reported as;

- *Bayesian* WISCA value;
- Number of appropriate antibiotic choices (1^st^ quantile *Bayesian* WISCA value greater than 0.5); and
- *Bayesian* WISCA value in different ICP adherence groups and probabilistic differences.

### Overview of definitions and methodological steps followed

The study included four steps to meet the defined objectives. These were as follows:

Step 1: Data collection and report of prevalence.
Step 2: Calculations of *Bayesian* WISCA.
Step 3: Evaluation of appropriateness of antibiotic choices.
Step 4: Comparison of investigator-reported ICP adherence groups.

**Step-1:** Data was collected from an annual surveillance study of HAUTIs in urology departments. HAUTI were defined according to the Center for Disease Control (CDC) criteria [14]. Analysis was limited to the European cohort only. The prevalence of HAUTIs, their causative pathogens and susceptibility profiles towards antibiotics suitable for UTIs were obtained from this data [7, 13].

**Fig 1:**
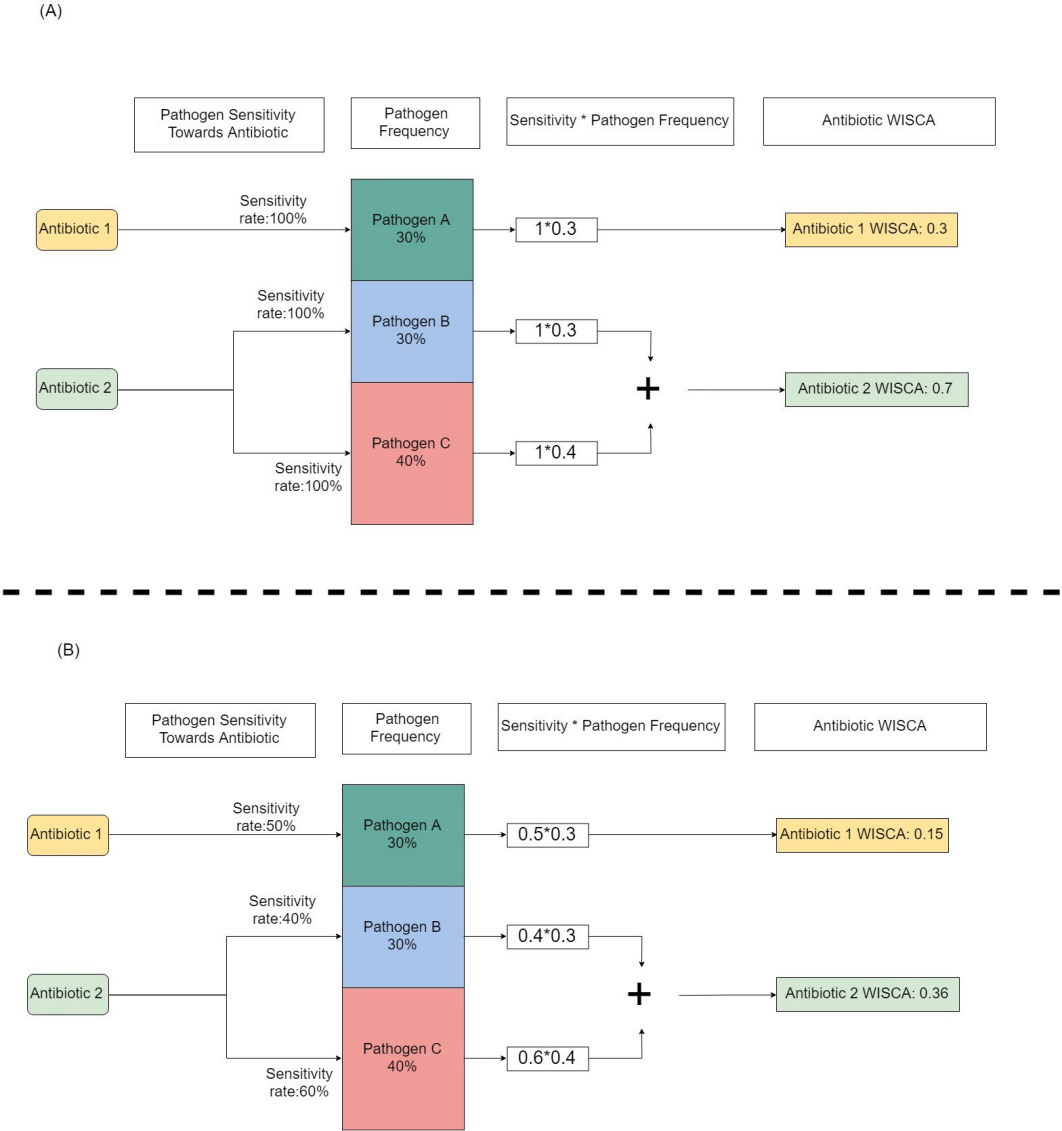
A hypothetical example of calculating WISCA. For purposes pf simplification, we assume that an infection can only be caused by three pathogens (A, B and C). Their frequency in a surveillance study was 30%, 40% and 30% for pathogen *A*, *B* and *C* respectively. For this disease there are two available antibiotics and Antibiotic 1 can only act against pathogen *A* and antibiotic 2 can act towards pathogen *B* and *C*. In Fig 1A an “all sensitive” environment is illustrated with the WISCA values for each antibiotic. In Fig 1B, an environment with “some resistance” is illustrated with the accompanied impact on the WISCA values. WISCA is a compound measure of surveillance derived causative pathogen frequency and susceptibility.

**Step-2**: The probability of an antibiotic to cover the causative pathogens of the infectious disease was calculated using the WISCA framework. Coverage means causative pathogens are sensitive to the selected antibiotic. An illustration simplified of the principle is provided in Fig 1. WISCA was calculated by using the *Bayesian* approach, which allowed inclusion of prior information (annual sequential data) and rare pathogens.

**Fig 2:**
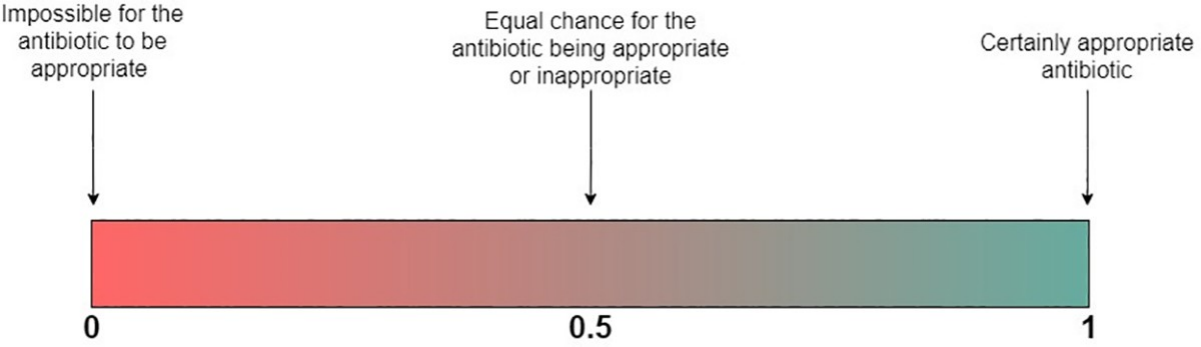
Description of WISCA properties. WISCA is a probability for an antibiotic to be appropriate for a certain infectious disease. Probabilities lie between 0 to 1. Therefore, if an antibiotic has a probability of 1 it is certain to cover all possible causative pathogens whilst these pathogens are all sensitive towards the antibiotic. This information is derived from surveillance of a population and therefore it cannot make inference to the possible effectiveness for an individual, which requires consideration of pharmacokinetic variables.

**Step-3:** Antibiotic choices were classified as either appropriate or inappropriate for empirical use in HAUTIs. This was determined using the *Bayesian* WISCA value. An antibiotic choice was classified as appropriate if the chances of coverage exceeded its chances of not providing coverage. The approach is illustrated in Fig 2. The list of antibiotic agents recommended for use in HAUTIs was obtained from international guidelines [15]. In total 18 antibiotics choices (single:10 & combinations:8) were evaluated (S1 Table).

**Step-4:** The impact of ICPs on appropriate antibiotic choices was estimated by means of simulation methods, which were reported in terms of probabilities. This approach allows accounting for the uncertainty of each antibiotic choice’s coverage chances.

### Step 1: Data collection—Annual surveillance of HAUTIs in urology departments

The GPIU study is a point prevalence study of HAUTIs in urology departments, conducted annually since 2003 [13]. The study protocol was amended in 2005 and data onwards was used for consistency. The study is open for recruitment from urology departments on the same 13 days of November each year. Ethical approval for the study was left at the departments discretion on the study years from 2003 to 2006 [16]. In 2007 a central ethical approval from Giessen University ethical committee in Germany was obtained. Data provided by participating departments was completely anonymized. The study is endorsed by the European Association of Urology (EAU) and participating centers are provided a certificate of participation by the EAU.

On the study day, all patients in the participating urology wards are screened for HAUTI and surgical site infections (SSI). CDC criteria are used to determine an infection episode (S1 Appendix) [14]. Participating departments are required to provide information regarding their hospital and urology department conditions and adherence to ICP. Clinical information including culture specimens is collected from patients with an episode of HAUTI or SSI. Antibiograms are measured at the recruiting center laboratories. An antibiotic measured as sensitive on an antibiogram (*in vitro)* is regarded as an appropriate choice for the episode of infection [17].

From 2005 to 2015, 365 urology departments screened 22,263 cases and reported 1,468 (prevalence:6.6%) symptomatic HAUTIs with microbiological proof of infection. Summary of patient acquisition according to STROBE guidance is provided in S1 Fig. Highest number of registries was from European departments (n:1,181–80%), followed by Asia (n:165–12%), Africa (n:42–3%) and Americas (n:36–2%). In 44 (3%) cases the geographical source of data was missing. We used the data provided by the European centers for analysis due to insufficient annual sample size of other regions. Countries that provided data and the number of centers from each country are reported in S2 Table.

### Step 2: Bayesian WISCA calculation

**Fig 3:**
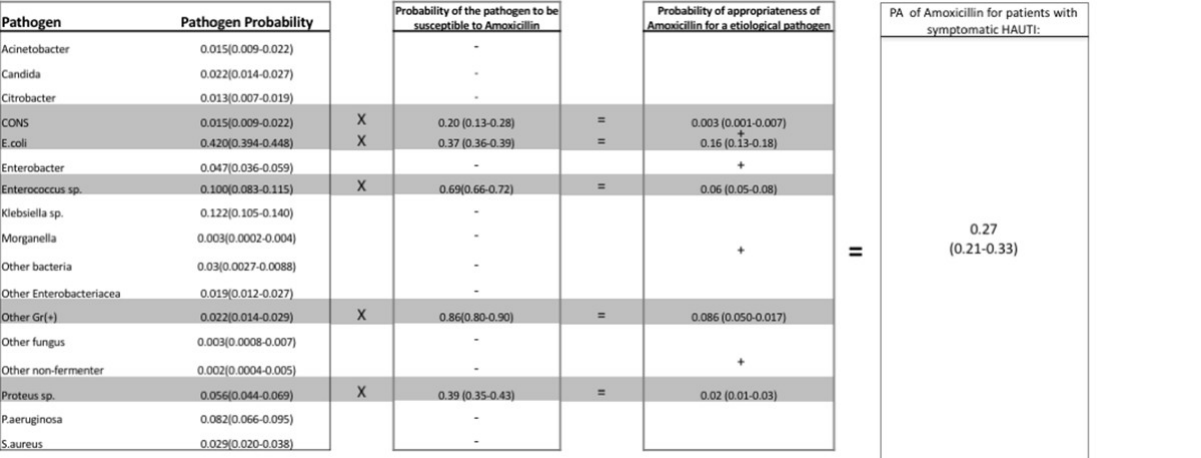
A practical example of calculating the WISCA for Amoxicillin in patients with symptomatic HAUTIs. Values are given as means and 95% uncertainty range. Amoxicillin can act against wild type *E*.*coli*, *Proteus* spp, *Enterococcus* spp, Coagulase (-) *Staphylococcus* (CONS) and other gram(+) isolates. The probability that each of these isolates cause a symptomatic HAUTI within the GPIU data is; 0.42, 0.056, 0.1, 0.015 and 0.022 respectively. The probability that each of these etiological pathogens are susceptible to Amoxicillin is; 0.37 (0.36–0.39), 0.39 (0.35–0.43), 0.69 (0.66–0.72), 0.20 (0.13–0.28) and 0.86 (0.80–0.90) respectively. The probability that Amoxicillin can cover each respective pathogen is; 0.16 (0.42*0.37), 0.02 (0.057*0.39), 0.06 (0.1*0.69), 0.003 (0.015*0.20) and 0.086 (0.022*0.86). By adding these probabilities up, the WISCA of Amoxicillin in HAUTIs is calculated to be 0.27.

The WISCA approach was adopted within the Bayesian framework [5, 6]. This represents the probability of an antibiotic to cover the causative pathogens for the surveyed infection. The analytical approach is provided in detail within S1 Appendix [6]. Briefly two parameters are used to calculate WISCA of an antibiotic: (i) probability of etiological pathogens; and (ii) probability of each etiological pathogen to be susceptible towards the studied antibiotic. WISCA is calculated by: (a) first multiplying the probability of each etiological pathogen with the corresponding probability of sensitivity of the studied antibiotic; and then (b) by adding those obtained probabilities. To obtain the probability distribution of the WISCA we use the GPIU prevalence data to simulate 1000 samples. A simplified example of WISCA calculation is provided in Fig 3.

Mainly we have two cores of calculations. The probabilities of etiological pathogens are sampled through the Dirichlet distribution (p_1_,…,p_n_), where p_j_ is the probability of pathogen (j). The probability of an antibiotic choice to be sensitive to a certain pathogen is sampled through the Beta distribution (sensitive, resistant). In both types we employ hierarchical modelling techniques on the GPIU data from the preceding two years of each studied year to calculate the informative priors. This is then combined with the raw data to obtain the posterior distributions for each studied year and calculate the *Bayesian* WISCA. A sensitivity analysis was conducted to determine the impact of the assumption of coding intermediate as resistant in the data set.

### Step 3: Appropriate empirical antibiotic choices for HAUTIs

Empirical treatment of patients with HAUTIs means that treatment is carried out in the absence of culture results to guide treatment. Therefore, it is important to distinguish between appropriate and inappropriate antibiotic choices. The empirical antibiotic choice will be appropriate if two conditions are satisfied. These are;

- The antibiotic should cover the causative pathogens; and
- The causative pathogen covered should be susceptible to the selected antibiotic choice.

The *Bayesian WISCA* identifies these two conditions and was used to classify antibiotic choices (Fig 2) as appropriate or inappropriate. HAUTIs are complex infections and initial empirical antibiotic treatment must be timely and effective. The consequences of failure of treatment can be dire. Therefore, the risk of failure should be kept as low as possible whilst also aiming to avoid antibiotic choices with high collateral damage.

In this study we sought to determine the number and characteristics (single agent vs multiple agent) of antibiotic choices that are appropriate for empirical treatment with the lowest possible risk of failure. In Fig 2 the principle of using the *Bayesian* WISCA value has been summarized. A threshold of 0.5 for the *Bayesian* WISCA was used to determine the appropriate antibiotic choices. In other words, a *Bayesian* WISCA above 0.5 represent greater chances of the antibiotic choice to provide coverage.

**Fig 4:**
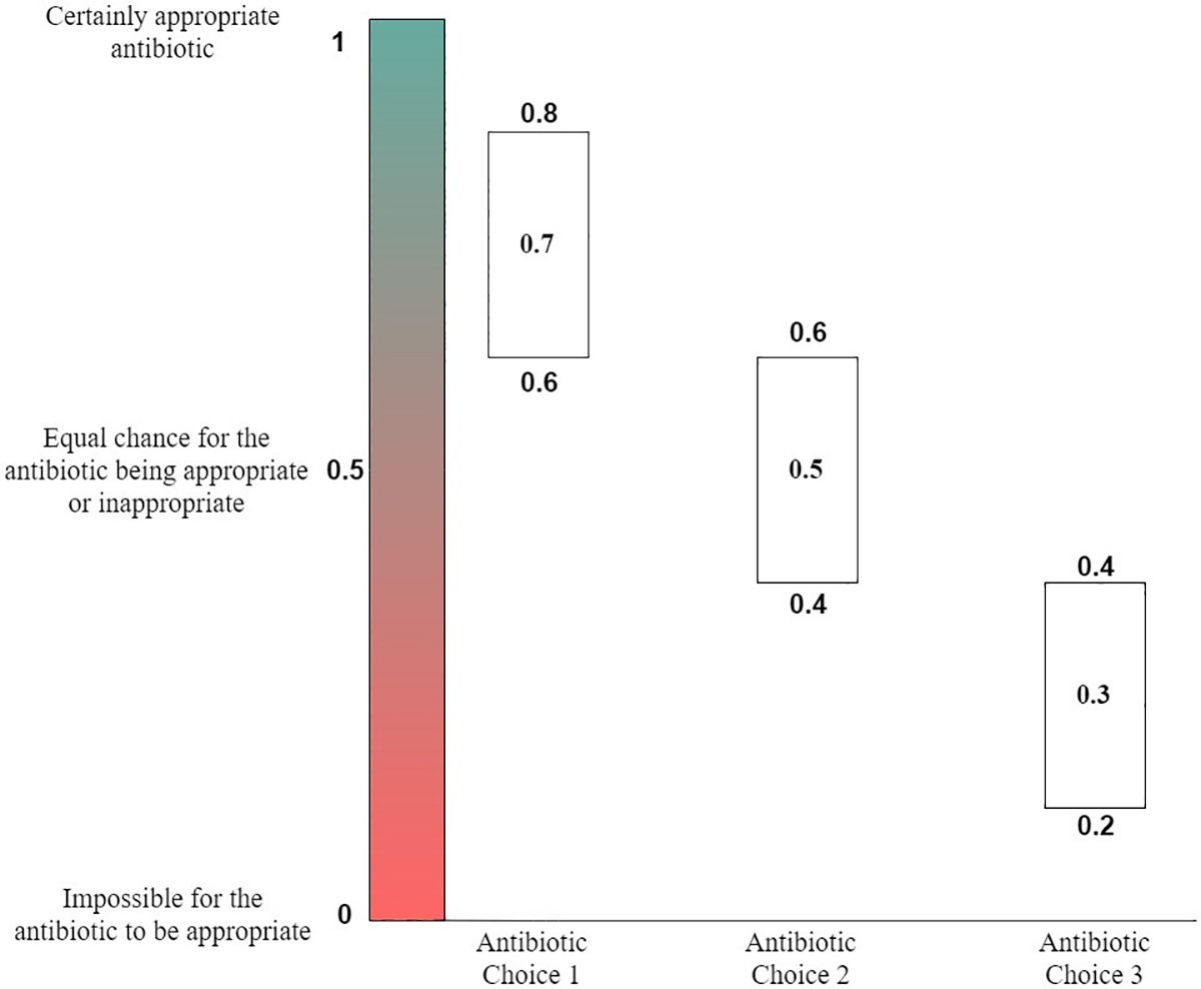
Example of how to estimate appropriateness of antibiotic choices. Antibiotic choices (single or multiple agents) are classified as appropriate or inappropriate. This is achieved by utilizing the Bayesian WISCA value for each choice. A threshold of 0.5 for the Bayesian WISCA value was selected. An antibiotic choice with a Bayesian WISCA value greater than 0.5 means that the chance of coverage exceeds the chance of not providing coverage. The interquartile range of the Bayesian WISCA was used to determine the appropriate antibiotics. This helped in accounting for the uncertainty level of each antibiotic choice to classify as appropriate or not. In the examples given antibiotic choice 1 has a mean Bayesian WISCA of 0.7 and 1st IQR value is 0.6. Since all simulated outcomes with this choice remain above 0.5 it is classified as appropriate. The second antibiotic choice presented has an IQR extending below 0.5 and despite a mean Bayesian WISCA of 0.5 is determined to be equivocal. The third antibiotic choice is classified as inappropriate since all the IQR values is below 0.5.

While a *Bayesian* WISCA threshold of above 0.5 helps for classification, utilizing the mean value alone cannot represent the uncertainty of the estimate in a useful way. The *Bayesian* WISCA estimate uncertainty is the range of values it can achieve and is represented using the interquartile range (IQR). To account for the estimate uncertainty and derive more robust decisions the IQR was used to establish thresholds. This is briefly illustrated in Fig 4.

Antibiotic choices with an estimated WISCA IQR below 0.5 are categorized as inappropriate antibiotics. Their chances of appropriateness are less than inappropriateness within the IQR of simulated samples. On the contrary antibiotic choices with an estimated WISCA IQR above 0.5 represents chances of appropriateness greater than inappropriateness within the IQR of simulated samples. Hence, those choices are categorized as appropriate antibiotics. For antibiotic choices with an IQR that overlaps the probability of 0.5 we are uncertain with their appropriateness. A summary of this stratification is provided in Table 1. A further threshold analysis was conducted to calculate the estimated number of appropriate antibiotics if the threshold was to be increased from 0.5 at an increment of 0.1 until no antibiotics remained appropriate.

**Table 1:** Categorization of antibiotics according to the interquartile ranges of WISCA values.

WISCA IQR	Appropriateness vs. inappropriateness	Category
0–0.5	Appropriateness< inappropriateness within the IQR of simulated samples	Inappropriate
0.5–1	Appropriateness> inappropriateness within the IQR of simulated samples	Appropriate
IQR LB*<0.5 and IQR UB^*^>0.5	Appropriateness< | >inappropriateness within a group of the IQR of simulated samples	Equivocal

*LB: lower bound UB: Upper bound

### Step 4: Comparison of infection control policies

Adherence to two ICPs was reported by investigators. These are; catheter management policy and policy for regulated usage of prophylactic antibiotics in urology departments. Based on the reported ICP adherence, hospitals were stratified into three groups. These were;

- *Complete adherence*: adherence to both policies;
- *Partial adherence*: adherence to one policy amongst two; and
- *Absence of ICP adherence*: adherence to none of the two policies.

*Bayesian* WISCA values were estimated for these three categories. Probabilistic comparison was achieved with the inverse cumulative density function method to simulate 1000 WISCA samples for each policy. This is followed by comparing each sample between groups to determine the probability of the WISCA to be greater than the comparator. This process classifies each sample superior or not to the comparator for each antibiotic choice. A threshold of 75% is used as an acceptable level for deducing conclusions on the trends. For example, if the *Bayesian* WISCA values of an antibiotic in group *x* is greater than those of the same antibiotic in group *y* in 75% of the sampled cases, then the antibiotic shows a trend of superior appropriateness when applied in group *x* rather than in group *y*. Of note, conventional reporting of *p-*values was avoided within the *B*ayesian framework and estimates are presented with a degree of certainty [18, 19].

## Results

**Fig 5:**
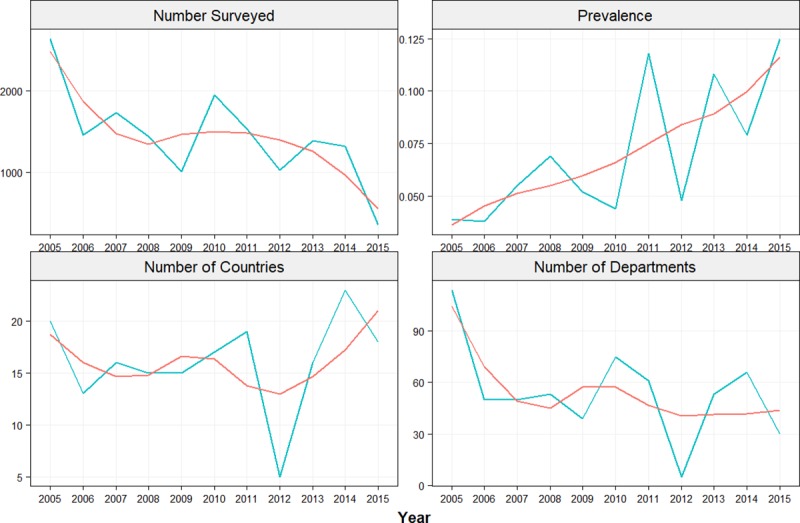
Annual number of patients screened, HAUTI prevalence, number of departments participating and number of countries in the GPIU study carried out in Europe.

The analyzed data consisted of 1,468 cases from Europe diagnosed with HAUTI (according to CDC criteria) from 2005 to 2015. The annual prevalence of HAUTIs in Europe ranged from 3.8% (2006) to 11.8% (2011). Urology departments from 29 countries across Europe provided data. The number of patients screened, departments and countries participating, and prevalence of HAUTIs with culture proven infection annually are presented in Fig 5.

The mean age of patients was 63.8 (IQR: 55–76) and gender ratio was 2:1 for male to female. The subgroups of diagnosis had a similar distribution (cystitis: 36.7%, pyelonephritis: 30.4% and urosepsis:32.8%).

### Antibiotic resistance rates

The sensitivity rates for *E*.*coli* ranged from 33% (Cefepime-2015) to 100% (Imipenem-except 2008) for single agents and from 75% (Ampicillin with Beta Lactamase Inhibitor (BLI) +Aminoglycosides in 2015) to 100% (all combinations in various years) for combinations. These rates for *E*.*coli* were obtained from a sample size ranging from 3 cases (Cefepime–2015) to 61 cases (Gentamicin-2014) (S3 Table). Sensitivity rates of pathogens against the antibiotic choices are provided in S2 Fig.

### Bayesian WISCA of antibiotics in HAUTIs

**Fig 6:**
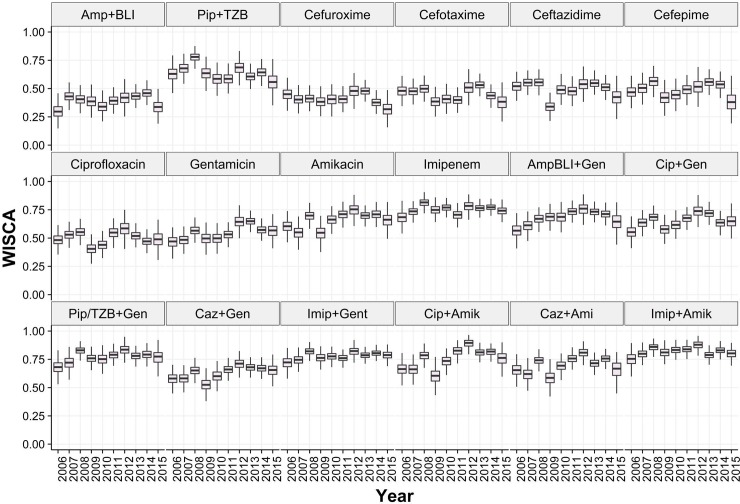
Estimated Bayesian WISCA (informative prior) of antibiotic choices in HAUTIs reported in European urology departments from 2006 to 2015.

The annual estimated mean *Bayesian* WISCA of antibiotics in HAUTIs of single agents ranged from 0.29 (IQR: 0.21–0.37) for Amp+BLI (2006) to 0.81(IQR:0.76–0.86) for Imipenem (2008) (Fig 6). The estimated mean *Bayesian* WISCA of all combination options was above 0.5. The lowest was estimated for Ciprofloxacin and Gentamicin combination in 2006 [mean:0.55 IQR:0.48–0.62] and 2009 [0.57 IQR:0.51–0.63]. Highest estimate was 0.87 [IQR:0.83–0.91] for Imipenem and Amikacin combination in 2009.

Annual trends were not apparent for the estimated Bayesian WISCA. The highest mean estimated *Bayesian* WISCA for single agents was for Imipenem in all consecutive years, which was above 0.7 in all instances. In all years except for 2015 there was at least one combination choice with an estimated Bayesian WISCA of greater than 0.8. Sensitivity analysis for the Bayesian WISCA did not impact the values (S3 Appendix)

### Appropriate empirical antibiotic choices for HAUTIs

The single agent antibiotic choices with a Bayesian WISCA IQR>0.5 were accepted as appropriate. Appropriate single agents were; Piperacillin+Tazobactam (2006–2014), Ceftazidime (2007,2008,2013), Cefepime (2008, 2012–2014), Gentamicin (2008, 2011–2014), Amikacin (2006–2008, 2010–2015), Imipenem (2006–2015) and Ciprofloxacin (2006, 2007, 2010, 2011). Throughout all study years Amoxicllin+BLI, Cefuroxime and Cefotaxime were categorized as inappropriate. Unlike the single antibiotic choices, all combination agents were classified as appropriate throughout the study years except for 2009 where we were uncertain with one combination choice.

The proportion of single agent antibiotics classified as appropriate (WISCA IQR>0.5) fluctuated throughout the years without an obvious trend (Table 2). Highest rates (>60%) were in 2008, 2012 and 2013. For the remaining years the proportion of appropriate single agents were 50% and below. We estimated a consistent proportion of uncertain antibiotics in terms of their appropriateness (10–30%).

**Table 2:** Proportion of antibiotics categorized as appropriate, inappropriate and equivocal in HAUTIs.

	Appropriateness	2006	2007	2008	2009	2010	2011	2012	2013	2014	2015
Single antibiotic agents	Inappropriate	40%	30%	20%	60%	50%	30%	20%	20%	30%	50%
	Appropriate	30%	50%	70%	20%	30%	50%	60%	60%	50%	20%
	Equivocal	30%	20%	10%	20%	20%	20%	20%	20%	20%	30%
Combination antibiotic agents	Inappropriate	0	0	0	0	0	0	0	0	0	0
	Appropriate	100%	100%	100%	87.5%	100%	100%	100%	100%	100%	100%
	Equivocal	0	0	0	12.5%	0	0	0	0	0	0

**Fig 7:**
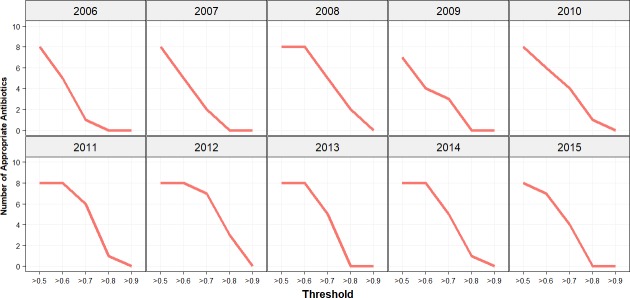
Appropriate antibiotics threshold analysis for single antibiotic choices. Incremental increases of 0.1 on the threshold from 0.5 onwards to determine appropriate antibiotics for single agent choices. On y-axis the number of appropriate antibiotics and on the x-axis the threshold value applied on the lower IQR is illustrated. Increase in threshold has gradually declined the number of appropriate antibiotics.

**Fig 8:**
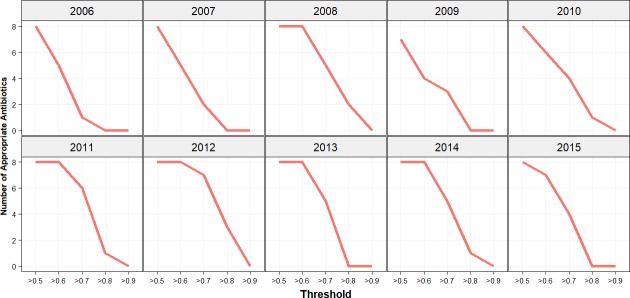
Appropriate antibiotics threshold analysis for combination antibiotic choices. Incremental increases of 0.1 on the threshold to determine appropriate antibiotics for combination agent choices. On y-axis the number of appropriate antibiotics and on the x-axis the threshold value applied on the lower IQR is illustrated. Increase in threshold has gradually declined the number of appropriate antibiotics.

Analysis of the established threshold was repeated with an increase of 0.1 increments. The results for single agent antibiotic choices are illustrated in Fig 7. Apart from 2006 and 2011 none of the antibiotics were appropriate at a threshold of 0.8. A similar impact on the decline in appropriate antibiotics was estimated for combination choices but at a threshold of 0.8 in a few study years there was a single agent remaining appropriate (Fig 8). In the years 2008, 2011, 2012, 2013 and 2014 the number of appropriate antibiotics remained unchanged at a threshold of 0.6 for the *Bayesian* WISCA.

### Impact of ICPs on antibiotic choices

**Fig 9:**
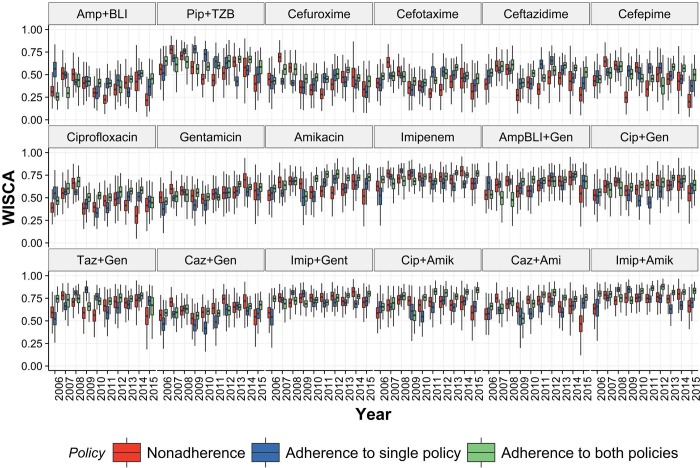
Estimates of Bayesian WISCA (with informative prior) of antibiotic choices per infection prevention policy in HAUTIs from urology departments reported in Europe from 2006 to 2015.

**Fig 10:**
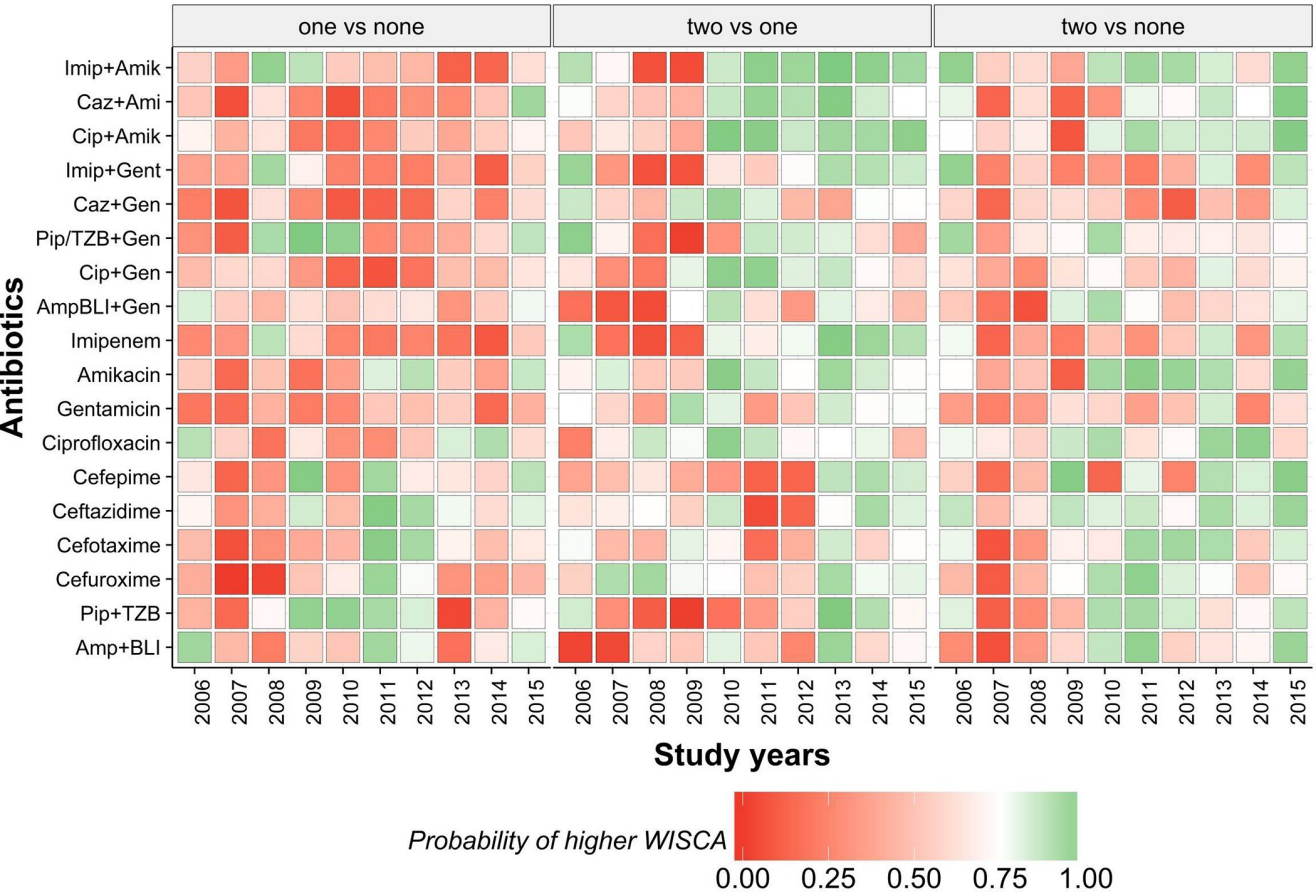
Probability that Bayesian WISCA of the antibiotic choice at the particular year is superior in the departments "x" compared with department "y" (departments are stratified according to the number infection control policy adherence).

The *Bayesian* WISCA values of antibiotics in departments with different ICP adherences are illustrated in Fig 9. The wide range of uncertainty in departments with absence of ICPs is of notice owing to low sample size (S4 Table). Proportion of antibiotics that were appropriate ranged between 33%-88%, 27%-83% and 61%-91% for departments with absence, partial adherence and complete adherence of ICPs respectively. An overall time trend is not apparent, and we used the sampled method to compare superiority of WISCA between departments (Fig 10).

Partial adherence vs. absence of ICPs: The estimated WISCA IQRs showed overlap in most time points in these two categories (Fig 9). We are uncertain if partial adherence provides an improved estimated WISCA compared to absence of ICP. The number of superior antibiotic choices in departments with partial ICP adherence compared to complete absence of ICP adherence ranged between 0 (2007–2008) to seven (39%) over the study years (Fig 10).

Complete adherence vs partial adherence: The number of superior antibiotic choices over the study years ranged between two (11%) (2007–2008) to 16 (88%) (2013) (Fig 10). Amongst eight combination agent choices except for Amp/BLI+Gentamicin and Ciprofloxacin+Amikacin all remaining options estimated Bayesian WISCA was superior in departments with complete adherence to ICPs (Fig 10).

Complete adherence vs. absence of ICP: Number of superior estimates of antibiotic choices ranged between 0 (2007–2008) to 13 (72%) (2015) (Fig 10). From 2008 onwards, the complete adherence group exhibited at least 4 (22%) (2014) antibiotic choices that were superior.

## Discussion

We evaluated the chances of coverage of empirical antibiotic choices through the *Bayesian* WISCA in European urology departments for HAUTIs defined by strict criteria (CDC) over a period of 10-years. We estimated that the mean *Bayesian* WISCA value for single agent antibiotic choices ranged from 0.29 (Amp+BLI in 2006) to 0.81 (Imipenem 2008). Combination of antibiotic choices showed an increase in the estimates. By further utilizing the analytic methods we were able to stratify the antibiotic choices as appropriate, inappropriate and equivocal for empirical treatment of HAUTIs. We estimated that half of the single antibiotic agents are inappropriate and for one third of them we are uncertain. In our estimates all combination agents remained appropriate throughout the study years. Our analytical approach has also demonstrated that self-reported adherence to a range of infection prevention policies coincides with higher number of antibiotic choices.

Chance of providing coverage (WISCA) is a relatively new concept for evaluating antibiotics by accounting for both frequency of HAUTI pathogens and their respective AMR [17, 20, 21]. To our knowledge there are no studies in the published literature that utilized this approach for HAUTIs. A study from Netherlands in 2012 used the method for complicated UTIs but lacked validated instruments to capture clinical information and characterize infections [22]. On the contrary, we studied a very specific population by using stringent criteria (CDC definitions) for the diagnosis of HAUTI. Therefore, we were able to distinguish asymptomatic bacteriuria and exclude these patients that constitute approximately one third of the population detected through screening of inpatients [16]. Ensuring a specific population is a key strength of our study but beyond that utilizing the WISCA in a surveillance study is limited as rare pathogens cannot be accounted for. To overcome this the *Bayesian* analytical approach was utilized, which to our knowledge was first used by *Bielicki J.A. et al [5].* In their study, the *Bayesian* WISCA was used to select appropriate empirical antibiotics for blood stream infections in a pediatric population. However, they used a non-informative prior, which means they didn’t account for prior year surveillance data. In our study we used robust validated methods (hierarchical modelling) to include the prior year data (informative prior) when calculating the *Bayesian* WISCA value [6]. In brief, inclusion of the *Bayesian* approach into WISCA with an informative prior has facilitated to obtain probabilities of coverage for antibiotic choices with their respective level of uncertainty.

The *Bayesian* WISCA values of the studied antibiotics showed fluctuations over the 10-year period but lacked a clear time trend. We further assessed the antibiotic choices by establishing cut-off values to determine if an antibiotic would be an appropriate choice for empirical treatment. Similarly, this approach also did not demonstrate a time trend in the proportion of appropriate antibiotic choices. However, this finding should be interpreted with caution as we are uncertain with at least 20% of single agent choices. We also conducted a threshold analysis, whereby the threshold was gradually increased at an increment of 0.1. This demonstrated that in several years the threshold could be increased to 0.6 without a decrease in the number of appropriate antibiotics. This exploratory analysis is particularly important as it can be utilized in local surveillance data to establish the optimal decision for appropriate antibiotic lists whilst controlling for risk of failure.

It should be emphasized that by classifying an antibiotic as inappropriate in this study we are not suggesting them to be redundant and we encourage their utility in the presence of culture and sensitivity results if proven to be sensitive. Optimal antibiotic selection approaches are still to be discovered, but in our study, we confirmed that combination options improve the *Bayesian* WISCA proposing a plausible strategy for complicated, severe HAUTIs such as urosepsis. For example, the mean *Bayesian* WISCA estimate of Amp+BLI alone ranged between 0.29 (IQR:0.26–0.34, 2006) to 0.46 (IQR:0.43–0.49, 2014) and for Gentamicin alone ranged from 0.46 (IQR:0.43–0.50, 2006) to 0.65 (IQR:0.62–0.67, 2013). The combination of these two antibiotics lead to a substantial increase in the mean *Bayesian* WISCA estimate ranging from 0.56 (2006, IQR:0.53–0.61) to 0.76 (2012, IQR:0.72–0.79). This combination choice, like other Beta-lactam and Aminoglycoside combinations, is estimated to have a similar chance of coverage with broad spectrum antibiotics such as Imipenem and Piperacillin/Tazobactam that should be conserved as much as possible in the face of increasing antibiotic resistance. The examples provided illustrate the need for careful interpretation of the obtained estimates. Moreover, the *Bayesian* WISCA can facilitate finding effective strategies for selecting appropriate antibiotics for empirical treatment. We would also stress out the importance of making better choices of empirical treatments with the *Bayesian* WISCA. If one opts to obtain an absolute certainty of complete coverage of pathogens with empirical treatment the *Bayesian* WISCA (estimate of 1 with no uncertainty) then multiple agents and classes of antibiotics would be combined. This would, however, defeat the purpose of antibiotic stewardship and optimization of choices.

As part of our work we carried out an exploratory analysis of the impact of ICPs on appropriate antibiotics through the *Bayesian* WISCA tools. ICPs can reduce the need (less infection episodes) and demand (responsible use) for antibiotics [23]. The policies evaluated in our study were adherence to urinary catheter care and regulation of antibiotic usage for prophylaxis. The first policy is expected to decrease catheter associated UTIs hence reducing the need for antibiotics. The second is associated with a decline in the demand for antibiotics. We have estimated that compliance to both ICPs is necessary to achieve the benefits in terms of higher level of appropriate antibiotics (Fig 10). These findings highlight the importance of adhering to ICPs and the need for an extensive policy approach. Nevertheless, the estimates for the ICPs should be considered with caution since we lack detailed knowledge about the implementation and the strategies employed [24]. In our study investigators provided information on self-perceived adherence to the policies, which underline the need for a more detailed survey of this information. We therefore suggest that future studies should utilize the *Bayesian* WISCA framework to obtain comparable and reliable outcomes, which is one of the main difficulties of infection related policy studies. In our study, comparison of ICPs was carried out by utilizing probabilistic and simulation methods.

Despite the advantages of the novel approach we adopted in analyzing HAUTIs there are certain limitations that require attention. Firstly, the GPIU surveillance study gathers data from urology departments through an altruistic contribution. Therefore, insufficient sampling of the HAUTI population is of concern. However, departments contributing to this study do have a high level of awareness of AMR issues. Therefore, even if our results are based on an incomplete representation, its more likely to be the optimal section of the population. Secondly, only two ICPs were evaluated and unfortunately the local practice of these ICPs is not known. Thirdly, these estimates represent a sampled population in Europe at given time points and cannot be used to derive local empiric antibiotic policies. By pooling different hospital data, the inter-hospital heterogeneity has not been accounted for. We could have preferred to subgroup the samples of each hospital, but this would lead to very low sample sizes and unreliable estimates. Studies to assess inter-hospital heterogeneity as part of the *Bayesian* WISCA are required. Nevertheless, our study does provide an overall trend over a 10-year period in urology practice. Finally, we have not accounted for the geographical variation in Europe. This was due to insufficient data to represent certain areas and would generate uncertain results although the mean estimates would not change.

In conclusion, we were able to estimate that there was no clear time trend in chances of coverage of the antibiotic choices in European urology departments. Departments with self-reported adherence to ICPs have more antibiotic choices. Future studies should focus on determining the optimal selection of antibiotics for subgroups of HAUTIs. Moreover, the *Bayesian* WISCA approach adopted should be repeated in local surveillance studies to improve local policy recommendations.

## Supporting information

S1 Fig Patient case disposition from the GPIU for the current study reported according to the STROBE criteria. (DOCX)

S2 Fig Antibiotic resistance rates in Europe obtained from the GPIU study. (DOCX)

S1 Appendix Definitions of infections used in the Global Prevalence of Infections in Urology Study. (DOCX)

S2 Appendix Analytical methods. (DOCX)

S3 Appendix Sensitivity analysis results of the Bayesian WISCA. (DOCX)

S4 Appendix GPIU Investigators. (DOCX)

S5 Appendix References used in supporting information. (DOCX)

S1 Table Antibiotic choices evaluated for empirical treatment of HAUTIs in urology. (DOCX)

S2 Table **A. Number of departments from each European country per year.** (“x” refers to no registries from the country on the GPIU study year). **B. Number of patients surveyed and the prevalence of HAUTIS per year.**
(DOCX)

S3 Table Pathogens identified in cases with HAUTIs in the consecutive GPIU study years. (DOCX)

S4 Table Number of patients in departments with different infection control practices. (DOCX)
