# Supplementary material for: Appropriate empiric antibiotic choices in health care associated urinary tract infections in urology departments in Europe from 2006 to 2015: A Bayesian analytical approach applied in a surveillance study
Source: PLoS One. 2019 Apr 25;14(4):e0214710. doi: 10.1371/journal.pone.0214710 (PMC6483335; doi:10.1371/journal.pone.0214710)
Supplement: S1 Fig — (DOCX) [file pone.0214710.s001.docx]

# **S1 Fig. Patient case disposition from the GPIU for the current study reported according to the STROBE criteria.**

**
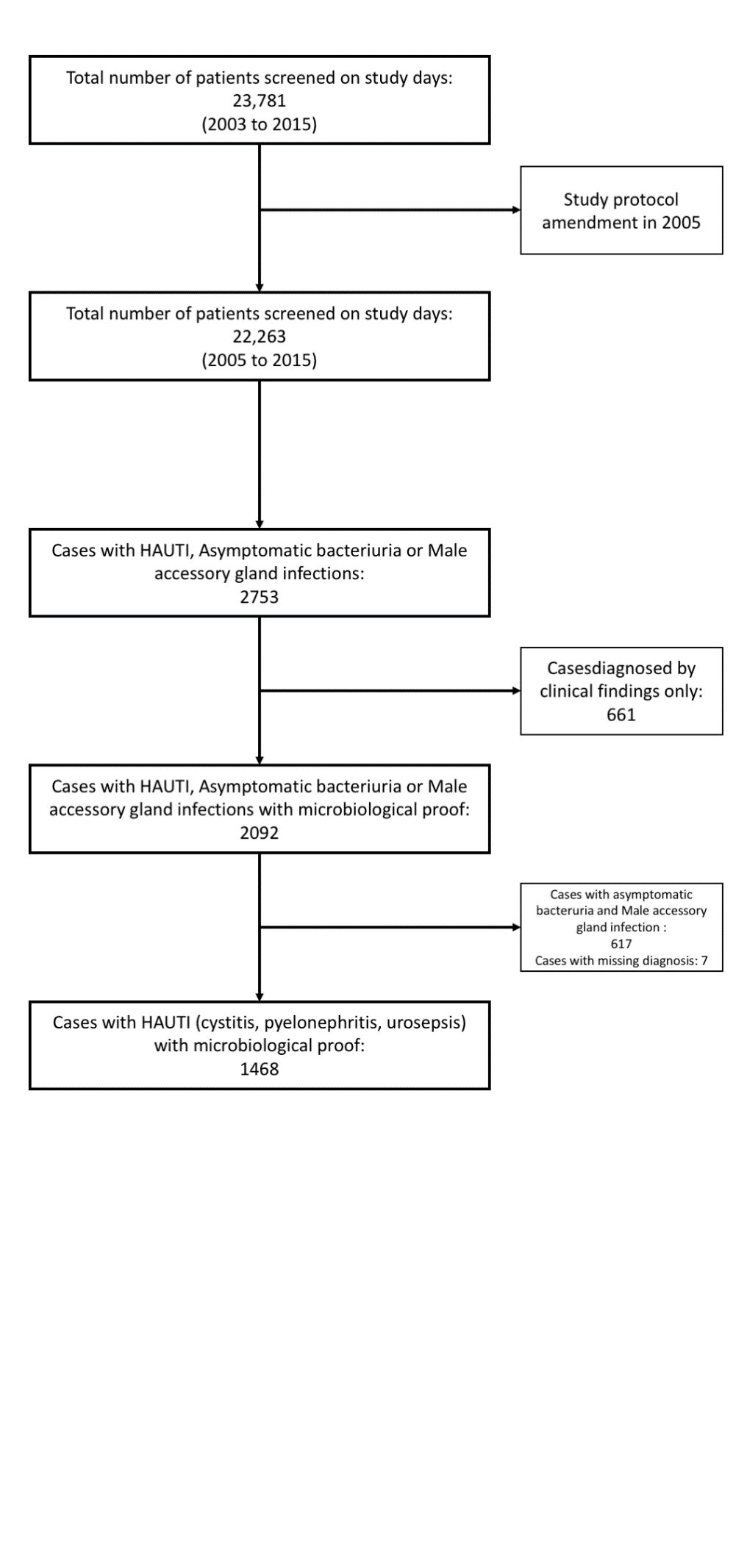
**

***patients from 2003 and 2004 study have been excluded due to changes in study forms and how hospital infection control programs were evaluated.**
