# Supplementary material for: Appropriate empiric antibiotic choices in health care associated urinary tract infections in urology departments in Europe from 2006 to 2015: A Bayesian analytical approach applied in a surveillance study
Source: PLoS One. 2019 Apr 25;14(4):e0214710. doi: 10.1371/journal.pone.0214710 (PMC6483335; doi:10.1371/journal.pone.0214710)

# **S2 Figs. Antibiotic resistance rates in Europe obtained from the GPIU study.**

S2 Figure A. Resistance rates of pathogens against single agent antibiotics in Europe on each studied year.


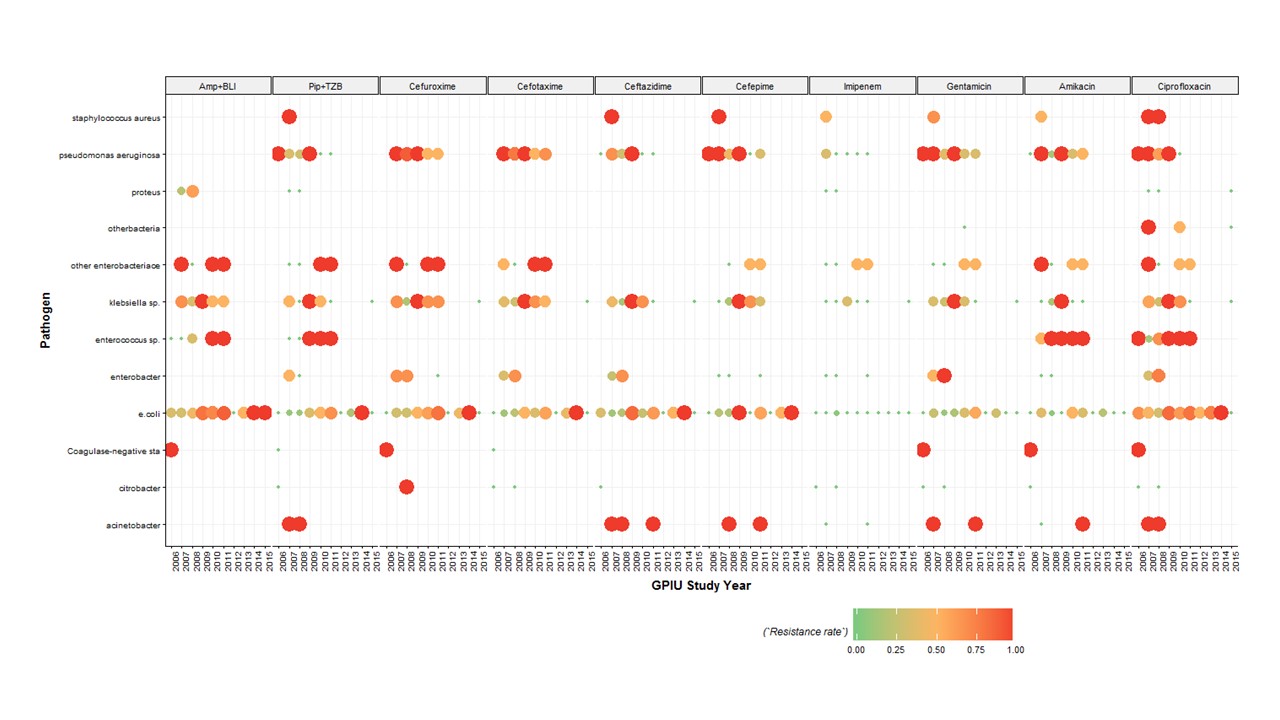


S2 Figure B. Resistance rates of pathogens against combination agent antibiotics in Europe on each studied year.


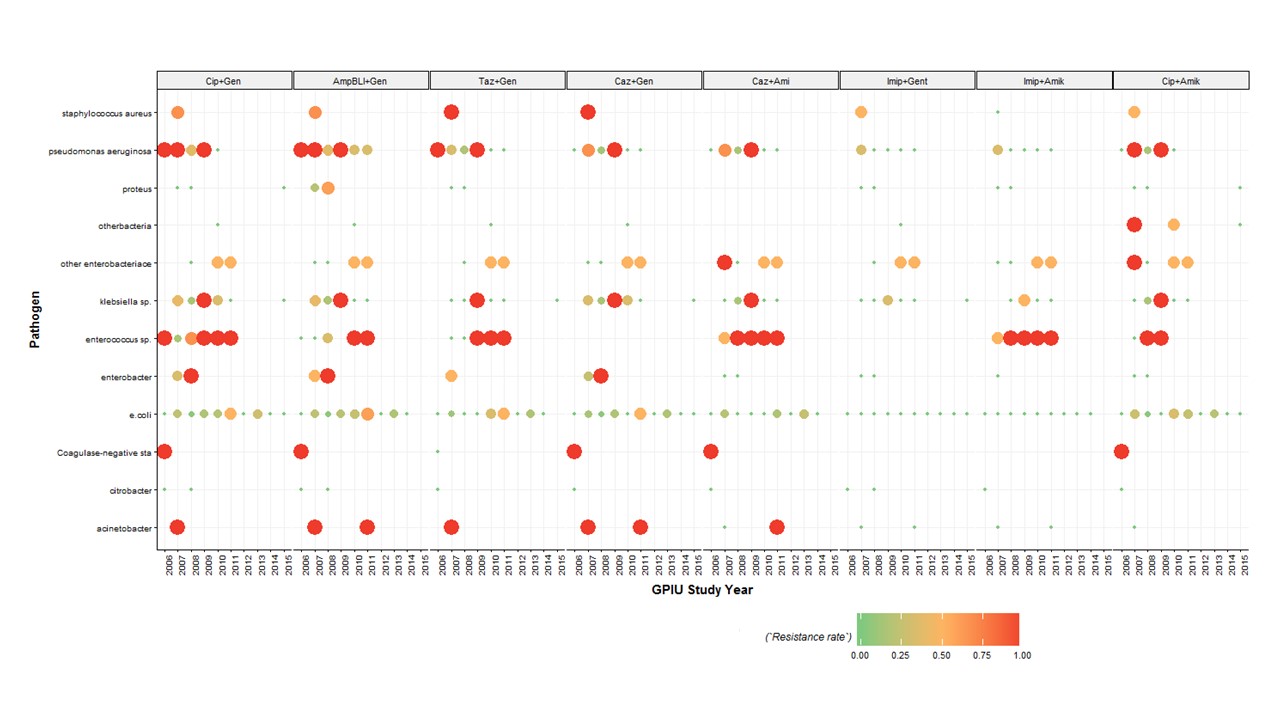

Supplement: S2 Fig — (DOCX) [file pone.0214710.s002.docx]
