# Supplementary material for: Appropriate empiric antibiotic choices in health care associated urinary tract infections in urology departments in Europe from 2006 to 2015: A Bayesian analytical approach applied in a surveillance study
Source: PLoS One. 2019 Apr 25;14(4):e0214710. doi: 10.1371/journal.pone.0214710 (PMC6483335; doi:10.1371/journal.pone.0214710)
Supplement: S1 Appendix — (DOCX) [file pone.0214710.s003.docx]

# **S1 Appendix: Definitions of Infections used in the Global Prevalence of Infections in Urology Study**

**Urinary Tract Infection**: Urinary tract infection includes symptomatic urinary tract infection, asymptomatic bacteriuria, and other infections of the urinary tract.

**Symptomatic urinary tract infection** must meet one of the following criteria:

1. One of the following: fever (>38°C), urgency, frequency, dysuria, or suprapubic tenderness AND a urine culture* of >= 10^5^ colonies/ml urine with no more than two species of organisms
2. Two of the following: fever (>38°C), urgency, frequency, dysuria, or suprapubic tenderness AND any of the following:
   1. Dipstick test positive for leukocyte esterase and/or nitrate.
   2. Pyuria (>=10 white blood cells (WBC)/ml or >=3 WBC/high-power field of unspun urine).
   3. Organisms seen on Gram stain of unspun urine
   4. Two urine cultures with repeated isolation of the same uropathogen^+^ with 10^2^ colonies/ml urine in nonvoided specimens..
   5. Urine culture with <=10^5^ colonies/ml urine of single uropathogen in patient being treated with appropriate antimicrobial therapy.
   6. Physician's diagnosis.
   7. Physician institutes appropriate antimicrobial therapy.
3. Patient <=12 months of age has one of the following: fever (>38°C), hypothermia (<37°C), apnea, bradycardia, dysuria, lethargy, or vomiting AND urine culture of 10^5^ colonies/ml urine with no more than two species of organisms.
4. Patient <=12 months of age has one of the following: fever (>38°C), hypothermia (>37°C), apnea, bradycardia, dysuria, lethargy, or vomiting AND any of the following:
   1. Dipstick test positive for leukocyte esterase and/or nitrate.
   2. Pyuria.
   3. Organisms seen on Gram stain of unspun urine.
   4. Two urine cultures with repeated isolation of same uropathogen with >=10^2^ organisms/ml urine in nonvoided specimens.
   5. Urine culture with <=10^5^ colonies/ml urine of a single uropathogen in patient being treated with appropriate antimicrobial therapy.
   6. Physician's diagnosis.
   7. Physician institutes appropriate antimicrobial therapy.

**Asymptomatic bacteriuria** must meet either of the following criteria:

1. An indwelling urinary catheter is present within 7 days before urine is cultured AND patient has no fever (>38°C), urgency, frequency, dysuria or suprapubic tenderness AND has urine culture of 105 organisms/ml urine with no more than two species of organisms.
2. No indwelling urinary catheter is present within 7 days before the first of two urine cultures with >=10^5^ organisms/ml urine of the same organism with no more than two species of organisms, AND patient has no fever(>38°C), urgency, frequency, dysuria, or suprapubic tenderness.

**Other infections of the urinary tract** (kidney, ureter, bladder, urethra, or tissues surrounding the retroperitoneal or perinephric spaces) must meet one of the following criteria:

1. Organism isolated from culture of fluid (other than urine) or tissue from affected site
2. An abscess or other evidence of infection seen on direct examination, during surgery, or by histopathologic examination
3. Two of the following: fever(>38°C), localized pain, or tenderness at involved site AND any of the following:
   1. Purulent drainage from affected site.
   2. Organism isolated from blood culture.
   3. Radiographic evidence of infection*.
   4. Physican's diagnosis.
   5. Physican institutes appropriate antimicrobial therapy.
4. Patient <=12 months of age has one of the followin: fever (>38°C), hypothermia (<37°C), apnea, bradycardia, lethargy, or vomiting AND any of the following:
   1. Purulent drainage from affected site.
   2. Organism isolated from blood culture.
   3. Radiographic evidence of infection.
   4. Physician's diagnosis.
   5. Physician instituted appropriate therapy.

*For urine specimens to be of value in determining whether a nosocomial infection exists, they must be obtained aseptically using an appropriate technique, such as clean catch collection, bladder catheterization, or suprapubic aspiration.

+Gram-negative bacteria of Staphylococcus saprophyticus.

**Urosepsis definitions and classisification used in the GPIU study*:**

| **Disorder** | **Definition** |
| --- | --- |
| Urosepsis (simple) | Activation of a systemic inflammatory response syndrome (SIRS) due to a urinary tract infection (UTI). This systemic response is manifested by two or more of the following conditions:  Temperature > 38°C or < 36°C.  Heart rate > 90 beats min.  Respiratory rate > 20 breaths/min or PaCO2 < 32mmHg (< 4.3kPa).  WBC > 12,000 cells/mm^3^ or < 4,000 cells/mm^3^ or ≥ 10% immature (band) forms. |
| Severe Urosepsis | Urosepsis associated with organ dysfunction, hypoperfusion or hypotension.  Hypoperfusion and perfusion abnormalities may include but are not limited to lactic acidosis, oliguria or an acute alteration of mental status. |
| Uroseptic Shock | Urosepsis with hypotension despite adequate fluid resuscitation along with the presence of perfusion abnormalities that may include, but are not limited to lactic acidosis, oliguria, or an acute alteration in mental status.  Patients who are on inotropic or vasopressor agents may not be hypotensive at the time that perfusion abnormalities are measured. |

*New sepsis definitions (Sepsis-3) have not been used as part of this study (Singer M, Deutschman CS, Seymour CW, Shankar-Hari M, et al. TheThird International Consensus Definitions for Sepsis and Septic Shock (Sepssis-3).JAMA. 2016 Feb 23;315(8):801-10. doi: 10.1001/jama.2016287)
