# Supplementary material for: Appropriate empiric antibiotic choices in health care associated urinary tract infections in urology departments in Europe from 2006 to 2015: A Bayesian analytical approach applied in a surveillance study
Source: PLoS One. 2019 Apr 25;14(4):e0214710. doi: 10.1371/journal.pone.0214710 (PMC6483335; doi:10.1371/journal.pone.0214710)
Supplement: S2 Appendix — (DOCX) [file pone.0214710.s004.docx]

# **Analytical Methods**

# **Obtaining probabilities and calculation of Bayesian WISCA of an antibiotic**

Hierarchical modelling to obtain prior probability distributions:

The prior probability distribution was obtained from the GPIU data of the preceding two years of studied year ^1^. Hierarchical modelling in the Bayesian framework can be used when data or its sources is organized in groups. In this context, the two study years before the year of interest are assessed as distinct groups that will be used to update our final probability estimate. The prior information ($\theta$) used to construct the conjugate posterior ($\omega$) will be assigned a prior distribution with unknown hyperparameters ($\varphi$). This hyperparameter will be assigned a distribution, which will be used to determine and update the conjugate posterior probability.

The Bioconductor package in “R” was used to obtain the hyperparameters (prior) for the pathogen distribution and the sensitivity ^2^.

Obtaining Probability of Etiological pathogens and antibiotic susceptibility:

The probability of each pathogen to be causative of a HAUTI is obtained from the GPIU prevalence data. The probabilities of etiological pathogens are sampled through the Dirichlet distribution (p_1(t)_,…,p_n_), where p_j_ is the probability of pathogen (j). Informative prior distribution (p_1(t-1 & t-2)_,…,p_n(t-1 & t-2)_) obtained using hierarchical modelling explained above.

Subsequently the conjugate posterior Dirichlet probability distribution (p_1(t-1 & t-2)_+ p_1(t)_,…,p_n(t-1 & t-2)_+p_n(t)_) was obtained.

Antibiotic susceptibility profile data in the GPIU was gathered as: resistant (r), intermediate and sensitive (s). Pathogen susceptibility profile was determined for 10 single agent antibiotics and 8 combination options, which are appropriate for use in UTIs (s-table-1). Each of these antibiotic options has a known antibacterial spectrum with a certain in-vitro and in–vivo activity. This list was obtained from European Committee on Antimicrobial Testing (EUCAST) consensus ^4^. For combination options, pathogens were considered sensitive if they were sensitive to at least one of the antibiotics. Possible synergistic, additional or antagonistic action of antibiotic combinations has not been taken into account.

Susceptibility profile (sensitive or not) was assumed to have a binomial distribution and the appropriate Beta-distribution was applied^5^. Prior distribution was assumed to be informative and was obtained as explained in step 1 (sensitive _(t-1 & t-2)_, resistant _(t-1 & t-2)_). Subsequently, the conjugate posterior distribution (sensitive _(t-1 & t-2)_+ sensitive _(t)_, resistant _(t-1 & t-2)_+ resistant _(t)_) was obtained .

For each antibiotic this was obtained for pathogens part of their susceptibility profile.

**Definition of Probability of appropriate antibiotic:**

For an infection there would be (m) number of causative pathogens. Running the Dirichlet distribution provides p_j_ probability of having pathogen j.

For this infection (n) number of antibiotics that can be used. Running the Beta distribution provides q_ij_ probability of the pathogen j being sensitive to Antibiotic i. Two inherent conditions apply for q_ij_ probability to be 0. First; the pathogen is intrinsically resistant to the antibiotic. Second, the pathogen is not part of the antibiotic susceptibility profile. Followed the total sampled probability of being sensitive to Antibiotic(A*i*) is

$$Probability of Appropriatness of Antibiotic\left( A_{i} \right)= \sum_{j=1}^{m} q_{ij}p_{j}$$

The parameter i runs over the values 1 to n.

**Computational approach:**

Probability of antibiotics being sensitive to a pathogen (q_ij_) is placed in a matrix Q = [q_ij_] and probability of pathogens being causative are placed as a column vector p = [p_j_]^T^. Followed by obtaining a sampled column vector for all antibiotics probability of appropriateness (PA) whose i entry gives the PA of Antibiotic_i_.

Application of the process and matrix multiplication:

$$PA=\left[ \begin{matrix} PA(A_{1}) \\ \vdots\\ PA(A_{n}) \end{matrix} \right] = \left[ \begin{matrix} \sum_{j=1}^{m} q_{1j}p_{j} \\ \vdots\\ \sum_{j=1}^{m} q_{nj}p_{j} \end{matrix} \right]= \left[ \begin{matrix} q_{11} & \cdots& q_{1m} \\ \vdots& \ddots& \vdots\\ q_{n1} & \cdots& q_{nm} \end{matrix} \right] \left[ \begin{matrix} p_{1} \\ \vdots\\ p_{m} \end{matrix} \right]=Qp$$
