# Supplementary material for: Appropriate empiric antibiotic choices in health care associated urinary tract infections in urology departments in Europe from 2006 to 2015: A Bayesian analytical approach applied in a surveillance study
Source: PLoS One. 2019 Apr 25;14(4):e0214710. doi: 10.1371/journal.pone.0214710 (PMC6483335; doi:10.1371/journal.pone.0214710)

# **S3 Appendix. Sensitivity analysis results for the calculation of *Bayesian* WISCA**

Calculation of the *Bayesian* WISCA assumed that all susceptibilities reported as intermediate were resistant. We carried out a sensitivity analysis where we provided a probability for the intermediate susceptibility to be resistant. This ranged from 0 to 1 at an increment of 0.1. After re-assigning the intermediate to resistant category we re-calculated the *Bayesian* WISCA. The results are provided in S3 Appendix Fig. The sensitivity analysis has revealed no impact on the *Bayesian* WISCA values.

S3 Appendix Fig. Sensitivity analysis results of the Bayesian WISCA. The assumption of categorizing intermediate susceptibility to resistant has been analyzed. This was achieved by providing a probability of coding intermediate as resistant. The probabilities attributed for recoding ranged from 0 to 1 with an increment of 0.1. For each probability of recoding the Bayesian WISCA values were re-calculated. On the x-axis the Bayesian WISCA values are provided and on the y-axis the probability of re-coding intermediate as sensitive is provided. Overall, the sensitivity analysis has illustrated no impact on the Bayesian WISCA values.


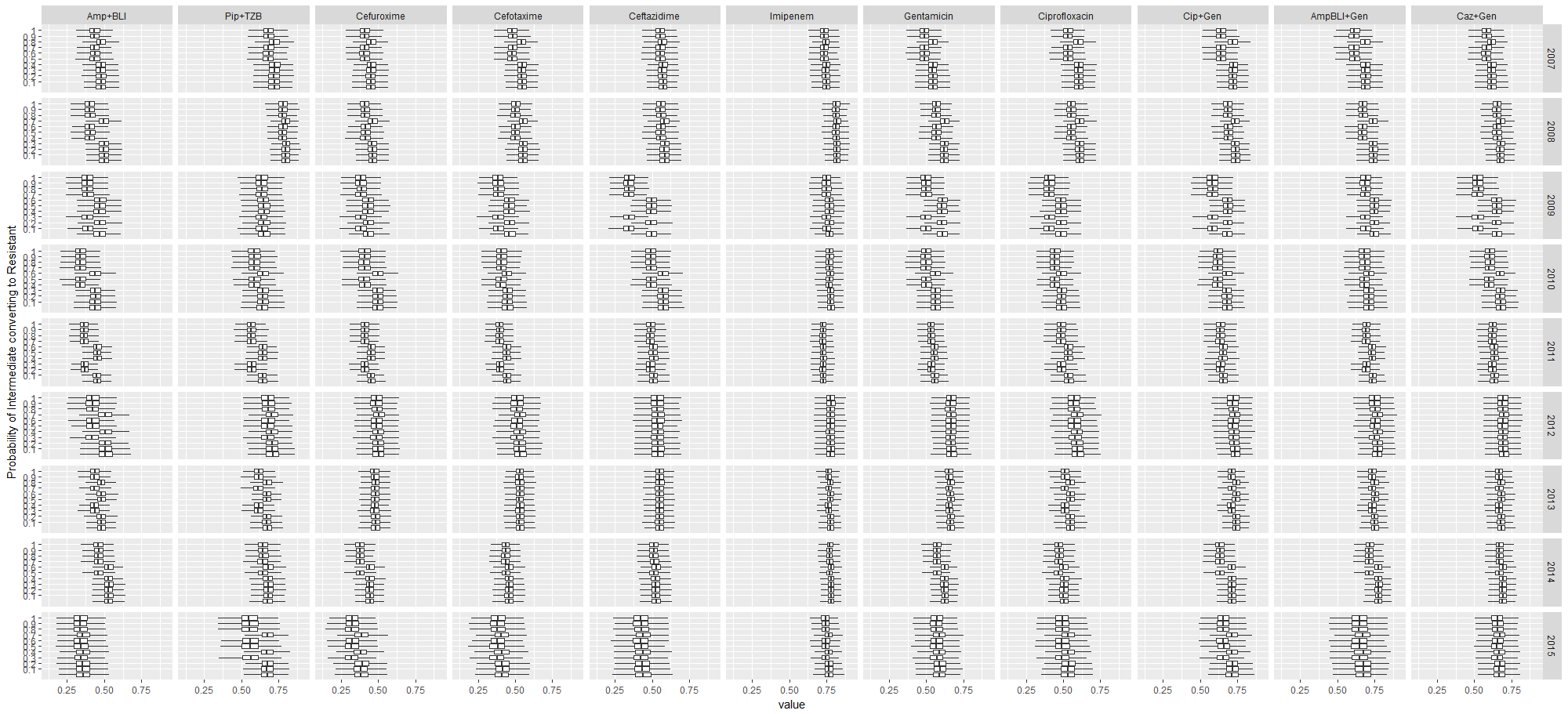

Supplement: S3 Appendix — (DOCX) [file pone.0214710.s005.docx]
