# Supplementary material for: Appropriate empiric antibiotic choices in health care associated urinary tract infections in urology departments in Europe from 2006 to 2015: A Bayesian analytical approach applied in a surveillance study
Source: PLoS One. 2019 Apr 25;14(4):e0214710. doi: 10.1371/journal.pone.0214710 (PMC6483335; doi:10.1371/journal.pone.0214710)
Supplement: S4 Appendix — (DOCX) [file pone.0214710.s006.docx]

**S4 Appendix. GPIU Study investigators.**

**From 2015 onwards**

| first_name | middle_name | last_name |
| --- | --- | --- |
| SINGEORZAN | | DORIN |
| Luka |  | Peneziackz |
| Margus |  | Krabi |
| Carlos A. | Ribeiro | Oliveira |
| Joao | Pimentel | Torres |
| Nuno | Sousa | Morais |
| Nelson |  | Morales |
| Barbara |  | Hermans |
| AndrÃ¡s |  | Magyar |
| Katarina |  | Grbesa |
| JosÃ© |  | Medina-Polo |
| Senad |  | Bajramovic |
| Frank | Ronald | Benzing |
| Sergii | Petrovich | Pasiechnikov |
| Mark | Fraser | Saxby |
| Carlos | Fernando | Andrade |
| THEODORE | | VOUDOUKIS |
| Duje |  | Rako |
| SERDAR |  | MADENDERE |
| LuÃ­sa | JerÃ³nimo | Alves |
| Roland |  | Donat |
| Emilio |  | Sacco |
| Florian |  | Wagenlehner |
| Catherine | Elizabeth | Philps Pereira |
| Medhat | Ahmed Mohamed | Elsayed |
| MUHAMMAD | SHERAZ | JAVED |
| Pedro | Daniel Oliveira Rocha | Costa |
| Haitham |  | Abdelmoteleb |
| SOUMYA |  | MISRA |
| Sergey | - | Shevchenko |
| Imre |  | Bode |
| Johan |  | Styrke |
| Andrei |  | Neculoiu |
| Abhijit | Shridhar | Dhale |
| TAYLAN |  | OKSAY |
| Branimir |  | Lodeta |
| Natalia |  | Sumerova |
| Akis | Alekz | Afoko |
| Tanja |  | Frank |
| REHA |  | GÄ°RGÄ°N |
| Tamara | Sergeevna | Perepanova |
| Pieter |  | D'hulst |
| Gokul Vignesh | | KandaSwamy |
| Ortwin |  | HeiÃŸler |
| Henrikki |  | Santti |
| Laia |  | Sos |
| Javier |  | Mayor de Castro |
| THEODORE | | VOUDOUKIS |
| Emad Eldin | | Mousa |
| konstantinos | | stamatiou |
| Fidel Tomas | Moyano | Manalaysay |
| FlÃ¡vio | Lobo | Heldwein |
| Basri |  | Cakiroglu |
| Ramona |  | Baldesi |
| francesco |  | chiancone |
| Masahiro |  | Matsumoto |
| Daniel |  | Schlager |
| Fiona Mei Wen | | Wu |
| Stephan |  | Miller |
| Edmund |  | Chiong |
| JAVIER | ANTONIO | HERRERA MUÃ‘OZ |
| Bart |  | Geboers |
| SÃ³nia | Afonso | Ramos |
| franck |  | bruyere |
| HUGO | OTAOLA | ARCA |
| Mircea | Valentin | Pirvut |
| Sataa |  | Sallami |
| anil |  | erkan |
| Maria-Fernanda | | Lorenzo-Gomez |
| Vasileios | I | Sakalis |
| Mihhail |  | Å½arkovski |
| Joachim |  | Hirschmann |
| Serenella |  | Monagas |
| Eshiobo |  | Irekpita |
| Ana-MarÃ­a | | Tapia Herrero |
| Mareike |  | Buch-Heberling |
| Tiago | Manuel | Ribeiro de Oliveira |
| Pedro |  | LÃ³pez Cubillana |
| Maria |  | Fiol Riera |
| Sven |  | Nikles |
| Christopher | Chee Kong | Ho |
| Adam |  | Fukasz |
| Sang Rak |  | Bae |
| Istvan |  | Buzogany |
| Matea |  | PirÅ¡a |
| MÃ¡rton |  | Oroszi |
| Lehel |  | Dr. PÃ©terfi |
| Yong-Gil |  | Na |
| kristin |  | rennesund |
| Levente |  | LÃ¡szlÃ³ |
| Ãgnes |  | Rosecker |
| Seung Ki |  | Min |
| EU CHANG | | HWANG |
| Woong Bin | | Kim |
| Seungsoo |  | Lee |
| mohammad | hassan | alhamad |
| Spahovic |  | Hajrudin |
| LILIANA |  | GARCES |
| Erik |  | Sagen |
| BegoÃ±a |  | Ballesta |
| Dr. Mahmoud | | Alnesr, FEBU |
| Matteo |  | Ferrari |
| Hin | Fan | Chan |
| Jean CÃ©drick | | Fouda |
| Yuriy |  | Brook |
| Mustafa |  | Kadihasanoglu |
| Philipp |  | Ganssmann |
| Manjeet |  | Kumar |
| Nikolaos | Vasilios | Manolakis |
| Sergio |  | Fumero Arteaga |
| HAJRUDIN | | SPAHOVIC |
| Aikaterini |  | Tsionga |
| Daniel |  | MacÃ­k |
| zilvinas |  | Venclovas |
| Milena |  | Taskovska |
| GONZALO | | GARCIA FADRIQUE |
| Georgios |  | Georgiadis |
| Ana MarÃ­a | | Tapia Herrero |
| Selcuk |  | Sarikaya |
| CHUYEN | LE | VU |
| Rick |  | Popert |
| Mauro |  | Ragonese |
| Dean |  | MarkiÄ‡ |
| Rita | Rodrigues | Fonseca |
| Tanja |  | Gschliesser |
| mustafa |  | vurucu |
| Hyunsop |  | Choe |
| Medhat | Ahmed Mohamed | Elsayed |
| Abdulmunem | Mohammed | AL Sadi |

## **Until 2015**

| Bachar |  | Zelhof | Preston | United Kingdom |
| --- | --- | --- | --- | --- |
| Luis | Rafael | Zegarra | Lima | Peru |
| Romuald |  | Zdrojowy | Warsaw | Poland |
| Sebastian |  | Zbrun | Bern | Switzerland |
| Andrey | Vladimirovich | Zaytsev | Moscow | Russia |
| Mihhail |  | Zarkovski | Tartu | Estonia |
| Gianpaolo |  | Zanetti | Milan | Italy |
| Morad | Abdallh | Zaki | Sohag | Egypt |
| K | Kuruvilla | Zachariah | Ashington | United Kingdom |
| Maher | Fawzi | Zabaneh | Amman | Jordan |
| Muhammad | Kabiru | Yusuf | Kano | Nigeria |
| Buhalov | Yuri | Yuri | Kyiv | Ukraine |
| Ilkan |  | Yuksel | Edirne | Turkey |
| Abbas |  | Yousefi Rad | Ankara | Turkey |
| Jeong | Jae | Young | Inchun | Korea, South |
| Ma |  | Yong | Shandong | China |
| Gul | Ruhsar | Yilmaz | Ankara | Turkey |
| Mustafa |  | Yildirim | Manisa | Turkey |
| Asif |  | Yildirim | Istanbul | Turkey |
| Wang |  | Yi | Shen Yang | China |
| Aydin |  | Yenilmez | Eskisehir | Turkey |
| Ozgur |  | Yaycioglu | Adana | Turkey |
| Ismet |  | Yavascaoglu | Bursa | Turkey |
| Stephen Shei- Dei |  | Yang | New Taipei City | Taiwan |
| Nguyen | Dinh | Xuong | Ho Chi Minh | Vietnam |
| Chao | Guan | Xu | Shiyan | Armenia |
| Yuan |  | Xiaoyi | Wuhan | China |
| Zhang |  | Xiangbo | Lanzhou | China |
| Fiona | Mei Wen | Wu | Singapore | Singapore |
| Eiling |  | Wu | Plymouth | United Kingdom |
| Eric | Roger | Wroclawski | Santo Andraja | Brazil |
| Slawomir | Pawel | Wozniak | London | United Kingdom |
| Ming Kui |  | Wong | W. P. Labuan | Malaysia |
| Gregory | Johann | Wirth |  | Switzerland |
| Joerg |  | Winkle | Aschaffenburg | Germany |
| Anika |  | Winkel | Recklinghausen | Germany |
| Florian | Thomas | Wimpissinger | Vienna | Austria |
| Simon |  | Williams | Derby | United Kingdom |
| Jan | Van Der | Wijk | STADSKANAAL | Netherlands |
| Thamara |  | Wijesuriya | Ragama | Sri Lanka |
| Jacqueline | ­­ | Wicht | Brandenburg | Germany |
| Veronica |  | Weterings | Breda | Netherlands |
| Martin |  | Westenfelder | Krefeld | Germany |
| Peter |  | Weib | Siegen | Germany |
| Madhav | Harihar | Waze | Panjim | India |
| Graham | Michael | Watson | Near Polegate | United Kingdom |
| Yi |  | Wang | Shengyang | China |
| Wei |  | Wang | Beijing | China |
| Mikala |  | Wang | Aarhus N | Denmark |
| Alexander | Arkadievich | Walsky | Ninsk | Belarus |
| Raphaela | Maria | Waidelich | Munich | Germany |
| Florian | Martin Erich | Wagenlehner | Giessen | Germany |
| Koichiro |  | Wada | Okayama | Japan |
| Vinka |  | Vukotic | Belgrade | Serbia and Montenegro |
| Chuyen |  | Vu Le | Ho Chi Minh | Vietnam |
| Le | Nguyen | Vu | Hanoi | Vietnam |
| Jaroslav |  | Vseticka | Jablonec Nad Nisou | Czech Republic |
| Theodore |  | Voudoukis | Aeghion | Greece |
| Nikolay | Andreevich | Vorobyov | Saint-Petersburg | Russia |
| Martin |  | Vorauer | Baden | Austria |
| Martin |  | Vorauer | Baden | Austria |
| Friedrich | Paul | Von Toggenburg | St. Gallen | Switzerland |
| Bjoern | Georg | Volkmer | Ulm | Germany |
| Thomas | Daniel | Vogt | Ergolding | Germany |
| Martin |  | Vogelsang | Ulm | Germany |
| Yannis |  | Vlamopoulos | Lausanne | Switzerland |
| Starodoubtsevan | Nadia | Vladimirovna | Pervouralsk | Russia |
| Maltsev | Andrey | Vladimirovich | Donetsk | Ukraine |
| Luchitskiy |  | Vitaliy | Kiyv | Ukraine |
| Jaspal | S | Virdi | Harlow,Essex | United Kingdom |
| Alexey | Dmitrievich | Vinokurov | Saint-Petersburg | Russia |
| Nataliya | Andreevna | Vinarova | Moscow | Russia |
| Brigitta |  | Villumsen | Holstebro | Denmark |
| Artsem |  | Viliukha | Minsk | Belarus |
| Angelo M. |  | Viggiano | Leon | Spain |
| Nguyen | Phu | Viet | Hanoi | Vietnam |
|  |  |  |  |  |
| Alberto |  | Vianello | Perugia | Italy |
| Tibor |  | Veszpremi |  | Hungary |
| Mr Prateek |  | Verma | Eastbourne | United Kingdom |
| Lisandro | Ignacio | Veliz | Buenos Aires | Argentina |
| Valia | Valentinova | Veleva | Sofia | Bulgaria |
| Muthu | Veeramani | Veeramani | Nadiad | India |
| Zoltan |  | Varga | Sigmaringen | Germany |
| Gabriel |  | Varga | Brno | Czech Republic |
| Marco |  | Varaldo | Genova | Italy |
| Paul |  | Van Wijk | Amsterdam | Netherlands |
| Jan | Paul | Van Haverbeke | Tielt | Belgium |
| Nathalie |  | Van Der Mee-Marquet | Tours | France |
| Michael | Rogier | Van Balken | Arnhem | Netherlands |
| Mstislav | Morozov | Valentinovich | Smolensk | Russia |
| Haluk |  | Vahaboglu | Izmit | Turkey |
| Markku | H | Vaarala | OYS | Finland |
| Nuray |  | Uzun | Istanbul | Turkey |
| Akylbek | Ch. | Usupbaev | Bishkek | Kyrgyzstan |
| Enzo |  | Usai | Cagliari | Italy |
| Juan |  | Uria | Vic | Spain |
| Suleyman | Erdinc | Unluer | Istanbul | Turkey |
| Dogan |  | Unal | Ankara | Turkey |
| Soner |  | Ulusoy | Istanbul | Turkey |
| Tomohiro |  | Ueda | Kyoto | Japan |
| Md Mohsin |  | Uddin | Dhaka | Bangladesh |
| Vasilios |  | Tzortzis | Larissa | Greece |
| Tahsin |  | Turunc | ADANA | Turkey |
| Bruce |  | Turner | London | United Kingdom |
| Levent | Niyazi | Turkeri | Istanbul | Turkey |
| Polat |  | Turker | Istanbul | Turkey |
| Cane | Dz | Tulic | Belgrade | Serbia and Montenegro |
| Muzaffar |  | Tukhtamishev | Tashkent | Uzbekistan |
| Andrea |  | Tubaro | Rome | Italy |
| Anton |  | Tsukanov | Omsk | Russia |
| Nadiya | Stepanivna | Tselyukh | Lviv | Ukraine |
| Petros |  | Tsafrakidis | Thessaloniki | Greece |
| Urszula | Grazyna | Trykozko | Warsaw | Poland |
| Andrej |  | Trusila | Minsk | Belarus |
| Tron |  | Tronsen | Levanger | Norway |
| Alberto |  | Trinchieri | Lecco | Italy |
| Yulia | Pavel | Trifonova | Kiev | Ukraine |
| Khac Linh |  | Tran Ngoc | Ho Chi Minh City | Vietnam |
| Nguyen | Van | Tran | Cantho | Vietnam |
| Svetia | Jordanov | Toujarov | Sofia | Bulgaria |
| Csaba |  | Toth | Debrecen | Hungary |
| Fettah |  | Tosun | Istanbul | Turkey |
| Gina | Marcela | Torres Zambrano | Madrid | Spain |
| Joãƒâ£O | Pimentel | Torres | Braga | Portugal |
| Jesus | Ignacio | Tornero | Murcia | Spain |
|  |  |  |  |  |
| Mohamed | Kotb | Tolba | Cairo | Egypt |
| Gokhan |  | Toktas | Istanbul | Turkey |
| Piero | Giovanni | Tognoni | Bogliasco-Genoa | Italy |
| Ilker |  | Tinay | Siirt | Turkey |
| Marco |  | Tiberi | Trento | Italy |
| Yahya | Tfeil | Tfeil | Nouakchott | Mauritania |
| Petr |  | Tesar | Praha 4 | Czech Republic |
| Peter |  | Tenke | Budapest | Hungary |
| Chu | Leong | Teh | Georgetown | Malaysia |
| Alaa | Abdallah | Tealab | Madinah Menwarra | Saudi Arabia |
| Maria Fe | Raymundo | Tayzon | Pasig City | Philippines |
| Meltem |  | Tasbakan | Izmir | Turkey |
| Msasanobu |  | Tanimura | Kochi-City | Japan |
| Neelam |  | Taneja | Chandigarh | India |
| Zafer |  | Tandogdu | Istanbul | Turkey |
| Kazushi |  | Tanaka | Kobe | Japan |
| Toomas |  | Tamm | Tallinn | Estonia |
| Arjana |  | Tambic Andrasevic | Zagreb | Croatia |
| Perepanova | Sergeevna | Tamara | Moscow | Russia |
| Nador |  | Tamas | Budapest | Hungary |
| Kaisar | Ali | Talukder | Dhaka | Bangladesh |
| Satoshi |  | Takahashi | Sapporo | Japan |
| Koichi |  | Takahashi | Onga Gun | Japan |
| Hamid | Reza | Tajari | Gorgan | Iran |
| Mehrdad |  | Tahami | Urmia | Iran |
| Ali |  | Taghizade Afshari | Urmia | Iran |
| Rahim | Razavi | Taghavi | Mashhad | Iran |
| Ferenc | - | Torzosk | G. | Hungary |
| Ervin |  | Szentgyorgyi | Vac | Hungary |
| Tamas |  | Szeberenyi | Gyula | Hungary |
| Andras |  | Szathmari | Miskolc | Hungary |
| Istvan |  | Szalay | Szeged | Hungary |
| Zoltan |  | Szabo | Kecskemet | Hungary |
| Clare |  | Sweeney | Aberdeen | United Kingdom |
| Joumana | Issa | Sunna | Amman | Jordan |
| Paul Anthony | Lugue | Sunga | Quezon City | Philippines |
| Fredrik |  | Sunden | Helsingborg | Sweden |
| Vesna | D | Suljagıc | Belgrade | Serbia and Montenegro |
| Branislav | Lazar | SUBOTIC | Brig | Switzerland |
| Nazareno |  | Suardi | Milan | Italy |
| Sergiy | Petrovych | Styopushkin | Dnipropetrovsk | Ukraine |
| Viveka | Diana | Strock | Goteborg | Sweden |
| Miroslav | Milorad | Stojadinovic | Kragujevac | Serbia and Montenegro |
| Ingunn |  | Steingrimsdottir | Reykjavãƒâ­k | Iceland |
| Dan |  | Stefanescu | Bistrita | Romania |
| Sotir |  | Stavridis | Skopje | Macedonia, The Former Yugoslav Republic of |
| Vladimir |  | Startsev | St.-Petersburg | Russia |
| Lesnikov | Vladimir | Stanislavovich | Donetsk | Ukraine |
| Maria | Ines | Staneloni | Buenos Aires | Argentina |
| Roman |  | Stanek | Opava | Czech Republic |
| Konstantinos |  | Stamatiou | Pireas | Greece |
| Pia |  | Stallenberg | Amsterdam | Netherlands |
| Annabelle |  | Stainier | Yvoir | Belgium |
| Christopher |  | Springer | Vienna | Austria |
| Annabel |  | Spek | Meiningen | Germany |
| Bjãƒâ¶Rn |  | Specht | Stuttgart | Germany |
| Hajrudin |  | Spahovic | Sarajevo | Bosnia and Herzegovina |
| Philipp | Julian | Spachmann | Regensburg | Germany |
| Petros |  | Sountoulides | Veria | Greece |
| Michelangelo |  | Sorrentino | Napoli | Italy |
| Jãƒâ¶Rg |  | Sommer | Lohne | Germany |
| Gyorgy | - | Solt | Harlow | United Kingdom |
| Thong |  | Sok Hean | Phnom Penh | Cambodia |
| Maja |  | Sofronievska Glavinov | Skopje | Macedonia, The Former Yugoslav Republic of |
| Mustafa |  | Sofikerim | Kayseri | Turkey |
| Doddy | M. | Soebadi | Surabaya | Indonesia |
| Chavdar | Krumov | Slavov | Sofia | Bulgaria |
| Aliaksandr |  | Sivets | Minsk | Belarus |
| Liubov | Alexandrovna | Sinyakova | Moskow | Russia |
| Rachhpal | S | Singh | Amritsar | India |
| Avinash | Kumar | Singh | Sofia | Bulgaria |
| Dorin |  | Singeorzan | Miercurea Ciuc | Romania |
| Orhun |  | Sinanoglu | Istanbul | Turkey |
| Battisti |  | Simon | La Roche Sur Yon Cedex 9 | France |
| Adrian | Diego | Simoes | Canterbury | United Kingdom |
| Ricardo | Pereira E | Silva | Lisboa | Portugal |
| Yoram | Itchak | Siegel | Zerifin | Israel |
| Marcin |  | Sieczkowski | Gdansk | Poland |
| Maksim |  | Sidorenko | Kiev | Ukraine |
| Babu Vijayakumar |  | Sidharaju | Salem | India |
| Alexander | Vladislavovich | Shuliak | Kiev | Ukraine |
| Mitra |  | Shodjai-Baghini | Vienna | Austria |
| Takehiko |  | Sho | Kitakyushu | Japan |
| Dimiter | Grigorov | Shishkov | Plovdiv | Bulgaria |
| Bongsuk |  | Shim | Seoul | Korea, South |
| Katsumi |  | Shigemura | Kobe | Japan |
| Alexey | Alexandrovich | Shevyrin | Ivanovo | Russia |
| Amir | Hossein | Sharafi | Urumia | Iran |
| Sergey | Nikolaevich | Shamrayev | Donetsk | Ukraine |
| Hassan | Sayed | Shaker | Cairo | Egypt |
| Mimoza | Ekrem | Shaipi | Presevo | Serbia and Montenegro |
| Syed | Ali | Shahzad | SLOUGH | United Kingdom |
| Garnik |  | Shahbazyan | Yerevan | Armenia |
| Nexhat | Emin | Shabani | Gjilan/Kosovo | Albania |
| Stavros | Fotios | Sfoungaristos | Karpathos | Greece |
| Musteba |  | Sevil | Afyon | Turkey |
| Joanne | Slyth | Serrano Uribe | Alcala De Henares | Spain |
| Alexander | Alexandrovitch | Seriogin | Moscow | Russia |
| Maroun |  | SERHAL | Beirut | Lebanon |
| Anup |  | Sengupta | Bury St. Edmunds | United Kingdom |
| Yavor | Petrov | Semerdzhiev | Sofia | Bulgaria |
| Mehmet | Bulent | Semerci | Izmir | Turkey |
| Oscar |  | Selvaggio | Matera | Italy |
| Ilker |  | Seckiner | Gaziantep | Turkey |
| Dr..Joseph | Philipraj | Sebastian | Gangtok | India |
| Alessandro |  | Sciarra | Rome | Italy |
| Bernhard | Michael | Schwindl | Weiden | Germany |
| Martin |  | Schoenthaler | Freiburg | Germany |
| Stefan |  | Schoeler | Mãƒâ¼Nchen | Germany |
| Ludvik |  | Schnitzer | Praha 4 | Czech Republic |
| Marcus |  | Schenck | Essen | Germany |
| Markus |  | Schãƒfer | Coburg | Germany |
| Markus |  | Schãƒâ¤Fer | Coburg | Germany |
| Sabrina |  | Schaeffer | Garmisch-Partenkirchen | Germany |
| Markus |  | Schãƒâ¶Nberger | K. | Germany |
| Anthony | J | Schaeffer | Chicago | United States |
| Roberto | Mario | Scarpa | Orbassano | Italy |
| Ariel | Gustavo | Scafuri | Fortaleza, Ceara | Brazil |
| Mark | Fraser | Saxby | Stoke-On-Trent | United Kingdom |
| Savvas | Tryfon | Savvaidis | Argos | Greece |
| Orlin |  | Savov | Nuremberg | Germany |
| Muppidi |  | Satyavani | Bandar Seri Bagawan | Brunei |
| Karen |  | Sarkisyan | Saint-Petersburg | Russia |
| Mehrdad |  | Sareh | Bucharest | Romania |
| Rosanna | Tubo | Santillan | Makati City | Philippines |
| Sandro | Danilo | Sandri | Magenta | Italy |
| Gecs |  | Sandor | Veszprãƒâ©M | Hungary |
| Susanna | Malin | Sandberg | Karlstad | Sweden |
| Murat |  | Samli | Afyon | Turkey |
| Hassan | Mikhael | Saloum | Riyadh | Saudi Arabia |
| Ahmed |  | Salman | Lahore | Pakistan |
| Sataa |  | Sallami | Tunis | Tunisia |
| Hosni | Khairy | Salem | Cairo | Egypt |
| Mohammad |  | Salehi | Rasht | Iran |
| Leonardo | Mose | Salame | Cefalu (Palermo) | Italy |
| Morshed | Ali | Salah | Al Wakra | Qatar |
| Vasileios | I | Sakalis | Thessaloniki | Greece |
| Radhia |  | Saidi | Monastir | Tunisia |
| Kapil |  | Sahnan | Gloucestershire | United Kingdom |
| Tayfun | - | Sahinkanat | Kahramanmaras | Turkey |
| Hayrettin |  | Şahin | Diyarbakir | Turkey |
| Erkin |  | Saglam | Istanbul | Turkey |
| Ahmed | S | Safwat | Assiut | Egypt |
| Refat | Abdelsamie | Sadeq | Zagazig | Egypt |
| Nourkhoda |  | Sadeghifard | Ilam | Iran |
| Emilio |  | Sacco | Roma | Italy |
| Brookman-Amissah |  | Sabine | Weiden | Germany |
| Sherif | Helmy | Saafan | Cairo | Egypt |
| M | M | S | Fayoum | Egypt |
| Emile |  | Rwamasirabo | Kigali | Rwanda |
| Andras |  | Rusz | Giessen | Germany |
| Annebeth |  | Ruiter | Nieuwegein | Netherlands |
| Robert | Raimund | Rudolph | Kirchheim | Germany |
| Levente |  | Rosztãƒâ³Czy | Baja | Hungary |
| ÃƒâGnes |  | Rosecker | Szeged | Hungary |
| Basilisk, | E | Rompis | Thessalonians | Greece |
| Imre |  | Romics | Budapest | Hungary |
| Alfonso | J. | Rodriguez-Morales | Trujillo | Venezuela |
| Viviana | M. | Rodriguez | Buenos Aires | Argentina |
| Cruz | N. | Rodriguez | Caracas | Venezuela |
| Raul | Nunes | Rodrigues | Angra Do Heroismo | Portugal |
| Rafael |  | Rodri­gues-Patraja | Madrid | Spain |
| Grãƒâ©Goire |  | Robert | Bordeaux | France |
| Torsten |  | Rith | Erlangen | Germany |
| Slobodan | Petar | Ristovski | Skopje | Macedonia, The Former Yugoslav Republic of |
| Pedro | M. | Rifakis | Caracas | Venezuela |
| Mehmet | Murat | Rifaioglu | Istanbul | Turkey |
| Hanna | Juliane | Richter | Garmisch-Partenkirchen | Germany |
| Carlos |  | Ribeiro Oliveira | Braga | Portugal |
| Theo | M | Reijke De | Amsterdam | Netherlands |
| Imre |  | Regos | Vac | Hungary |
| Harold | M | Red | Libertador San Martin | Argentina |
| Nouri |  | Rebai | Paris | France |
| Couti |  | Razvan | Cluj-Napoca | Romania |
| Dr | Syed Johar | Raza | Karachi | Pakistan |
| Raul |  | Raz | Afula | Israel |
| Mark |  | Rauthmann | Sigmaringen | Germany |
| Erika |  | Rauth | Pecs | Hungary |
| Deepak | Babu | Rauniyar | Dhulikhel | Nepal |
| Aso | Omer | Rashed | Sulaimani | Iraq |
| P.N. |  | Rao | Manchester | United Kingdom |
| Muhammad | Qamar Sarwar | Rana | Birmingham | United Kingdom |
| Anders |  | Ramsing | Vãƒâ¤Rnamo | Sweden |
| Rodrigo | Nuno Brito | Ramos | Lisboa | Portugal |
| Duje |  | Rako | Zagreb | Croatia |
| Lul |  | Raka | Prishtina | World |
| Hary Raj |  | Raja | Blackburn | United Kingdom |
| Syed | Imtiaz | Rahman | Newport | United Kingdom |
| Aino |  | Rãƒâμãƒâμm | Tallinn | Estonia |
| Muhammad |  | Rafique | MULTAN | Pakistan |
| Lili |  | Radulovic | Belgrade | Serbia and Montenegro |
| Sabri |  | Rachid | Casablanca | Morocco |
| Bryan | Ingemar | Qvick | Garmisch Partenkirchen | Germany |
| Ana Isabel | Linares | Quevedo | Madrid | Spain |
| Tahir | Uddin | Qazi | Kohat | Pacific Ocean |
| Wasim |  | Qasim | Dalian | China |
| Ralf |  | Pychynski | Bad Segeberg | Germany |
| Armin |  | Pycha | Bolzano | Italy |
| Praveen |  | Pushkar | New Delhi | India |
| Basuki | B | Purnomo | Malang | Indonesia |
| Tiziana |  | Puglisi | Ispica | Italy |
| Ignacio |  | Puche-Sanz | Granada | Spain |
| Petr |  | Prosvic | Nachod | Czech Republic |
| Jose-Vicente | Tablante | Prodigalidad | Quezon City | Philippines |
| Jose | Nestor | Procuna Hernandez | Mexico D.F. | Mexico |
| Domenico |  | Prezioso | Naples | Italy |
| Juan | Manuel | Poyato | Huelva | Spain |
| Mariela | Rosa | Pow-Sang | Lima | Peru |
| Tobias | Samuel | Pottek | Wedel | Germany |
| Martina |  | Porsch | Turnov | Czech Republic |
| Daniele |  | Porru | Pavia | Italy |
| Daniel | Hodade | Porav | Cluj-Napoca | Romania |
| Roberto |  | Ponchietti | Siena | Italy |
| Giorgio |  | Pomara | Pisa | Italy |
| Konstantinos |  | Polyzois | Manchester | United Kingdom |
| Hasan | S. | Pliev | Domodedovo | Russia |
| Alexei | Yurievich | Plekhanov | Saint-Petersburg | Russia |
| Pawel |  | Plaza | Lublin | Poland |
| Guido |  | Platz | Ruesselsheim | Germany |
| Mehmet | Mesut | Piskin | Konya | Turkey |
| Germar |  | Pinggera | Innsbruck | Austria |
| Mohan |  | Pillai | Blackburn | United Kingdom |
| Dirk |  | Piehler | Greifswald | Germany |
| Stefano |  | Picozzi | San Donato Milanese | Italy |
| David |  | Piccolotti | Lagosanto (FE) | Italy |
| Juan | Jose | Picazo | Madrid | Spain |
| Catherine | Elizabeth | Philps Pereira | Oslo | Norway |
| Joshua | Tarun | Phillips | Birmingham | United Kingdom |
| Yuriy | Yuryevich | Petrovskiy | Odessa | Ukraine |
| Georgios | Loizos | Petrikkos | Athens | Greece |
| John |  | Peters | London | United Kingdom |
| Melanie |  | Peter | Luebeck | Germany |
| Tunãƒâ« | P | Pervorfi | Prishtina | Serbia and Montenegro |
| Giacomo |  | Perugia | Rome | Italy |
| Dominik | Georg | Pernkopf | Vienna | Austria |
| Tamara | Sergeevna | Perepanova | Moscow | Russia |
| Miguel | Angel | Peredo | Mexico | Mexico |
| Dhelma | Isabel | Pellãƒâ­n | Caracas | Venezuela |
| Miguel | Angel | Pedrola | Venado Tuerto | Argentina |
| Rizky |  | Paukstadt | Hof | Germany |
| Antonio | L | Pastore | Terracina (LT) | Italy |
| Sergii | Petrovich | Pasiechnikov | Kiev | Ukraine |
| Jai | Pal | Paryani | Abu Dhabi | United Arab Emirates |
| Pawel |  | Parniewski | Lodz | Poland |
| Shin | Jae | Park | Daegu | Korea, South |
| Seung Chol |  | Park | Iksan | Korea, South |
| Christos |  | Papandreou | Arta | Greece |
| Anestis | G. | Papadopoulos | Ptolemayda | Greece |
| Vlad |  | Pantea | Gelsenkirchen | Germany |
| Ioannis |  | Panopoulos | Athens | Greece |
| Manoj | Kumar | Panigrahi | Berhampur | India |
| Shiv | Kumar | Pandian | Colchester | United Kingdom |
| Sanjay |  | Pandey | Mumbai | India |
| Juan |  | Palou | Barcelona | Spain |
| Ivan | S. | Palagin | Smolensk | Russia |
| Andras |  | Pãƒâ¡Czelt | Budapest | Hungary |
| Hakan |  | Ozveri | Istanbul | Turkey |
| Ahmet |  | Ozturk | Konya | Turkey |
| Firas |  | Ozoe | Chambery | France |
| Hakan |  | Ozkardes | Ankara | Turkey |
| Tayyar | Alp | OZKAN | ISTANBUL &Kocaeli | Turkey |
| Ilhan |  | Ozgunes | Eskisehir | Turkey |
| Muhammet | Fuat | Özcan | Ankara | Turkey |
| Alexander | Georgiev | Otzetov | Sofia | Bulgaria |
| Michael |  | Osei-Tether | Kumasi | Ghana |
| Alva | Damaris | Ortiz | La Libertad | El Salvador |
| Mazhar |  | Ortaãƒâ§ | Istanbul | Turkey |
| Samir | Shaaban | Orabi | Alexandria | Egypt |
| Chong Chien |  | Ooi | Selangor | Malaysia |
| Rahmi |  | Onur | Elazig | Turkey |
| Eng | Kwee | Ong | Barnstaple | United Kingdom |
| Daniel |  | Oliveira-Reis | Porto | Portugal |
| Kagan | Felixovich | Oleg | St.Persburg | Russia |
| Peter | Jochen | Olbert | Marburg | Germany |
| Taylan |  | Oksay | Isparta | Turkey |
| Athanassios |  | Oeconomou | Larissa | Greece |
| Yusuf | Vehbi | Ocak | Sanliurfa | Turkey |
| Gaga |  | Nutsubidze | Kutaisi | Georgia |
| Byron | F | Nunez Freile | Quito | Ecuador |
| Dmitry |  | Nitkin | Minsk | Belarus |
| Abolghasem |  | Nikfallah | Tehran | Iran |
| Heinz | Gerardo | Nicolai | Santiago | Chile |
| Dong | Le | Nguyen | Ho Chi Minh City | Vietnam |
| Roberto | Rossi | Neto | Essen | Germany |
| Elie | Ghannam | Nemr | Beirut | Lebanon |
| Hemant | Bhanudas | Nemade | Basildon | United Kingdom |
| Aleksei |  | Nelovkov | Tallinn | Estonia |
| Dana | Gabriela | Negru | Arad | Romania |
| Oktay |  | Nazlı | Izmir | Turkey |
| Stephen |  | Nazareth | Makati City | Philippines |
| Saygin |  | Nayman Alpat | Eskisehir | Turkey |
| Ala Eddin | Daud | Natsheh | Jerusalem | Israel |
| Matteo |  | Napoli | Trapani | Italy |
| Lukacs |  | Nandor | Kecskemet | Hungary |
| Djordje | Petar | Nale | Belgrade | Seychelles |
| Wataru |  | Nakamura | Tokyo | Japan |
| Haitham | Saeed | Nakad | Alswaida | Syria |
| Ali |  | Naghoni | Tehran | Iran |
| Akaki | A.N. | Nadareishvili | Tbilisi | Georgia |
| Samer |  | Nabolsi | Tulle | France |
| Ben Sorba |  | Nabil | Sousse | Tunisia |
| Kurt | G | Naber | Straubing | Germany |
| Yong-Gil |  | Na | Daejeon | Korea, South |
| Dmytro | Mykhailovich | Mykhailov | Kyiv | Ukraine |
| Nazim | - | Mutlu | KOCAELI | Turkey |
| Necmettin | Aydin | Mungan | Zonguldak | Turkey |
| Mehmet | Ugur | Mungan | Izmir | Turkey |
| Ismaila | A | Mungadi | Sokoto | Nigeria |
| Bakhadirkhanov | Mukhamed Zarif | Mukhamed Kabirkhonovich | Tashkent | Uzbekistan |
| Volker |  | Mueller-Mattheis | Duesseldorf | Germany |
| Kien | Alfred | Mteta | Moshi | Tanzania |
| Noreddin | Hamza | Msessa | Tripoli | Libya |
| Vladimir |  | Mozetic | Rijeka | Croatia |
| Javad | Nafchi | Moussavi | Szolnok | Hungary |
| Youssef |  | Moussa | Aleppo | Syria |
| Seyed Habibollah |  | Mousavi-Bahar | Hamadan | Iran |
| Emad Eldin | Khalid | Mousa | Sohar | Oman |
| Renato | Lains | Mota | LISBOA | Portugal |
| Giuseppe |  | Mostaccio | ESTE (PD) | Italy |
| Mohammad | Kazem | Moslemi | Qom | Iran |
| Saskia | Carmen | Morgenstern | Frankfurt Am Main | Germany |
| Rayo |  | Morfin | Guadalajara | Mexico |
| Manuel | F | Montesino | Eneriz | Spain |
| Myron | Sidonio | Monteiro | Bangor | United Kingdom |
| Nurbek | Kytaibekovich | Monolov | Bishkek | Kyrgyzstan |
| Gabriella |  | Mombelli | Magenta | Italy |
| Mayad | Nouma | Moktash | Najran | Saudi Arabia |
| Gholamreza |  | Mokhtari | Rasht | Iran |
| Tatyana | Nikolaevna | Moiseenko | Novosibirsk | Russia |
| Radman | Abdullah | Mohammed | Hodaidah | Yemen |
| Moudouni | Said | Mohammed | Marrakech | Morocco |
| Aza | A | Mohammed | Northampton | United Kingdom |
| Ahmed | Hedeia | Mohamed | Hurghada | Egypt |
| Mohammadreza |  | Moein | Yazd | Iran |
| Muhammad |  | Moazzam | Derby | United Kingdom |
| Lampros | Petros | Mitrakas | Larissa | Greece |
| Warli | Syah | Mirsya | Medan | Indonesia |
| Chen |  | Ming | Nanjing | China |
| Seung Ki |  | Min | Seoul | Korea, South |
| Stephan | M | Miller | Gelsenkirchen | Germany |
| Jose | Ignacio | Militello | Salta | Argentina |
| Karl | Joensen | Mikines | Herlev | Denmark |
| Vartolomei | M | Mihai Dorin | Ludus | Romania |
| Ilir | Iljaz | Miftari | Pristina | Albania |
| Jean-Claude |  | Miermont | Nimes | France |
| Ramunas |  | Mickevicius | Kaunas | Lithuania |
| Uwe | HG | Michl | Hamburg | Germany |
| Bernardino |  | Miãƒâ±Ana Lopez | Murcia | Spain |
| Naoufel |  | MIAADI | Rennes | France |
| Christian |  | Meyer | Hamburg | Germany |
| Denis | Abel | Mesa Borroto | Moron. Ciego De Avila | Cuba |
| Oguz |  | Mertoglu | Izmir | Turkey |
| Frank |  | Mennigen | Rheine | Germany |
| Violeta |  | Menendez Lopez | Sant Pere De Ribes | Spain |
| Massimo |  | Meneguolo | Belluno | Italy |
| Gustavo | Adolfo | Mendez | Posadas, Misiones | Argentina |
| Badaruddin | A | Memon | Khairpur | Pakistan |
| Michael | Dimitrios | Melekos | Larissa | Greece |
| Andreas |  | Meiãƒå¸Ner | Bonn | Germany |
| Jose |  | Medina-Polo | Madrid | Spain |
| Jan |  | Mecl | Liberec | Czech Republic |
| Javier |  | Mayor De Castro | Madrid | Spain |
| Alexander |  | Maykhir | Nizhny Novgorod | Russia |
| Ivan | Victorovich | Matveyeu | Vitebsk | Belarus |
| Deliu-Victor |  | Matei | Milan | Italy |
| Maria |  | Mastruzzo | Loma Hermosa. Pcia De Buenos Aires | Argentina |
| Massimo |  | Massarelli | Ivrea | Italy |
| Goran | S | Marusic | Novi Sad | Serbia and Montenegro |
| Dean |  | Markiã | Rijeka | Croatia |
| Chiara |  | Mariani | Pisa | Italy |
| Andreas | E.A. | Manseck | Ingolstadt | Germany |
| Aditya |  | Manjunath | Bristol | United Kingdom |
| Cristian | Nicolae | Manea | Cluj Napoca | Romania |
| Amar | Kumar | Manandhar | Varna | Bulgaria |
| Annika |  | Malmquist | Ystad | Sweden |
| Ercan |  | Malkoc | Istanbul | Turkey |
| Bartosz | Radoslaw | Malkiewicz | Wroclaw | Poland |
| Anton | I. | Maliavin | Ulyanovsk | Russia |
| Saidamin | Anvarovich | Makhsudov | Tashkent | Uzbekistan |
| Julia |  | Makarycheva | Samara | Russia |
| Michael |  | Maier | Rothenburg O. D. Tauber | Germany |
| Mahmoud | Farouk | Mahmoud Awad | Zagazig,Sharqia | Egypt |
| Benatta |  | Mahmoud | Oran | Algeria |
| Pattabiraman |  | Maheshkumar | Kings Lynn | United Kingdom |
| Ida | Soo-Fan | MAH | Hong Kong | Hong Kong |
| Vittorio-Gianpaolo |  | Magri-Perletti | Milano | Italy |
| Giuseppe |  | Magistro | Olching | Germany |
| Massimo | - | Maffezzini | Genova | Italy |
| Rafael | Velazquez | Macias | Mexico | Mexico |
| Ciaran | B | Lynch | Birmingham | United Kingdom |
| Oliver |  | Luzar | Bonn | Germany |
| Nicolae |  | LUPSASCA | Vitry Le Franãƒâ§Ois | France |
| Jens-Claudio |  | Lunz | Regensburg | Germany |
| Martin |  | Ludwig | Marburg | Germany |
| Qiao |  | Ludong | Beijing | China |
| Shing-Hwa |  | Lu | Taipei City | Taiwan |
| Kevin |  | Lu | Kaohsiung | Taiwan |
| Mikhail | * | Lozinskiy | Trnava | Slovakia |
| Andrea |  | Loreto | Roma | Italy |
| Miguel | Angel | Lopez-Aramburu | Miranda De Ebro (Burgos) | Spain |
| Andres |  | Lopez De Alda | Don Benito, Badajoz | Spain |
| Pedro |  | Lopez Cubillana | Murcia | Spain |
| Sofia | Pinheiro | Lopes | Loures | Portugal |
| Sudhir | Kumar | Lokwani | Bhopal,M.P. | India |
| Joyce |  | Loeffler | Nice | France |
| Branimir |  | Lodeta | Varazdin | Croatia |
| Michele |  | Lodde | Bolzano | Italy |
| Umberto |  | Locunto | Firenze | Italy |
| Chiwen |  | Lo | New Taipei City | Taiwan |
| Eva |  | Ljunggren | Lund | Sweden |
| Bosas |  | Liudas | Kaunas | Lithuania |
| Anna | AL | Liskova | Nitra | Slovakia |
| Gabriele |  | Lindenau | Vienna | Austria |
| Teng Lung |  | Lin | Taipei | Taiwan |
| Donghoon |  | Lim | Gwangju | Korea, South |
| Di | Pi | Li | Nasice | Croatia |
| Gabriel | Isaac | Levy Hara | Buenos Aires | Argentina |
| Fredrik |  | Levin | Stockholm | Sweden |
| Philippe |  | Lesprit | Crãƒâ©Teil | France |
| Peter | Hasan | Leonidovich | Moscow | Russia |
| Dr. Mohamed | Said | Leithy | Alexandria | Egypt |
| Jãƒâ¶Rg |  | Leifeld | Leer | Germany |
| Michael | Yu | Leh | Pasig | Philippines |
| Seung-Ju |  | Lee | Suwon | Korea, South |
| Sang Don |  | Lee | Yansan | Korea, South |
| Hyun-Rim |  | Lee | Buchon-Si | Korea, South |
| Elaine | Wei Yin | Lee | Harrogate, North Yorkshire | United Kingdom |
| Chang-Ho |  | Lee | Cheonan | Korea, South |
| Angus |  | Lecuona | Cape Town | South Africa |
| Aleksandar |  | Lazic | Belgrade | Serbia and Montenegro |
| Jan | Henrik | Laurick | Hagen | Germany |
| Stefano |  | Lauretti | Roma | Italy |
| Firstname | Middlename | Lastname | City | Country |
| Roland |  | Lang | Landshut | Germany |
| Daniel |  | Landau | Beer Sheva | Israel |
| Michael |  | Lamche | Vienna | Austria |
| Akos |  | Lakatos | Szentes | Hungary |
| Muhammad | Shahzad | Laghari | Scunthorpe | United Kingdom |
| Marãƒâ­a | Josãƒâ© | Lãƒâ³Pez Furst | Buenos Aires | Argentina |
| Vanda | Daniela | Lãƒâ³Pez | Caracas | Venezuela |
| Hanga |  | Laszlo Miklos | Papa | Hungary |
| Pasquale | Gianfranco | La Rosa | Giarre (CT) | Italy |
| Christian |  | La Hmann | Ulm | Germany |
| Espen |  | Kvan | Drammen | Norway |
| Marjan | Jovan | Kuzmanoski | Skopje | Macedonia, The Former Yugoslav Republic of |
| Dimitri |  | Kuzenko | Uelzen | Germany |
| Ainura | Zarylbekovna | Kutmanova | Bishkek | Kyrgyzstan |
| Fatih | Osman | Kurtulus | Istanbul | Turkey |
| Marcin |  | Kurant | Bydgoszcz | Poland |
| Shlomo | Mario | Kuntze | Richterswil | Switzerland |
| Hiromi |  | Kumon | Okayama | Japan |
| Ekaterina | V. | Kulchavenya | Novosibirsk | Russia |
| Alexander |  | Kugler | Marktredwitz | Germany |
| Shoji |  | Kudou | Chuo Yamanahsi | Japan |
| Aleksandra |  | Krznar | Celje | Slovenia |
| Alexander | Nikolaevich | Kruglov | Moskow | Russia |
| Fabian |  | Kronschnabl | Landshut | Germany |
| Christian |  | Kriegel | Leipzig | Germany |
| Benedikt |  | Kreiner | Berlin | Germany |
| W. |  | Kramer | Bad Soden | Germany |
| Nicole | Petra | Kraischits | Vienna | Austria |
| Margus |  | Krabi | Tallinn | Estonia |
| Tamas | Bognar | Kozma | Zalaegerszeg | Hungary |
| Zafer |  | Kozacioglu | Izmir | Turkey |
| Peter |  | Kovãcs | Eger | Hungary |
| Grigorios |  | Kousidis | ATHENS | Greece |
| Sotirios | Athanasios | Koukos | Ioannina | Greece |
| Rumen | Pachev | Kotsev | Pleven | Bulgaria |
| Serghiy | Mykolayovich | Kotsar | Mukachevo | Ukraine |
| Sergey | Vladislavovich | Kotov | Moscow | Russia |
| Shigeru |  | Kosugi | Tokyo | Japan |
| Alexander | Yrevich | KOROLEV | Saratov | Russia |
| Laszlo |  | Koranyi | Sopron | Hungary |
| Kãroly |  | Könvyes | Szombathely | Hungary |
| Angelis |  | Konstantinopoulos | Patras | Greece |
| Tome | Nik | Komani | Gjakova-Kosova | Serbia and Montenegro |
| Sanjay |  | Kolte | Nagpur | India |
| Ivan |  | Kolombo | Turnov | Czech Republic |
| Nikolay |  | Kolev | Pleven | Bulgaria |
| Dirk |  | Koglin | Ulm | Germany |
| Mikhail | Josefovich | Kogan | Rostov On Don | Russia |
| Lukas |  | Kocis | Poprad | Slovakia |
| Ramazan |  | Kocaaslan | Istanbul | Turkey |
| Roberto |  | Knez | Trieste | Italy |
| Jacques |  | Klein | Geneva | Switzerland |
| Hiroshi |  | Kiyota | Tokyo | Japan |
| Stiliana |  | Kiurkchieva | Sofia | Bulgaria |
| Attila |  | Kiss | Budapest | Hungary |
| Jacek |  | Kis | Krasnystaw | Poland |
| Sinan |  | Kirecci | Istanbul | Turkey |
| Pernille | Skjold | Kingo | Aarhus N | Denmark |
| Quinten | Michael | King | Palmerston North | New Zealand |
| Th |  | Kim | Seoul | Korea, South |
| Kiho |  | Kim | Gyeongju | Korea, South |
| Kang Sup |  | Kim | Seoul | Korea, South |
| Jung Hoon |  | Kim | Seoul | Korea, South |
| Jun-Mo |  | Kim | Bucheon | Korea, South |
| Jong Il |  | Kim | Busan | Korea, South |
| Hong Bin |  | Kim | Seongnam | Korea, South |
| Chul Sung |  | Kim | Korea | Korea, South |
| Mehmet |  | KILINC | Konya | Turkey |
| Margot | Martyna | Kieruj | Bergisch Gladbach | Germany |
| Iradj |  | Khosropanah | Rasht | Iran |
| Dinyar |  | Khazaeli | Ahvaz | Iran |
| Mhd | Mouayed | Khawajki | Damascus | Syria |
| Priya |  | Khanna | London | United Kingdom |
| Taskeen | Ahmad | Khan | Peshawar, NWFP | Pakistan |
| Shahid | Aziz Anwer | Khan | Redhill | United Kingdom |
| Farrukh | Kamal | Khan | Canterbury | United Kingdom |
| Faisal | Rauf | Khan | Newcastle Upon Tyne | United Kingdom |
| Rinat |  | Khammatov | Togliatti | Russia |
| Ismail | Mohamed | KHALAF | Cairo | Egypt |
| Karthi |  | Keyan | Cochin | India |
| Mehmet | Selcuk | Keskin | Ankara | Turkey |
| Gabor |  | Kerenyi | Eger | Hungary |
| Eray |  | Kemahli | Istanbul | Turkey |
| Nuri |  | Keimeroã„Å¸Lu | Bilecik | Turkey |
| Bastian |  | Keck | Erlangen | Germany |
| Fahimeh | - | Kazemi Rashed | Tabriz | Iran |
| Cevdet |  | Kaya | Istanbul | Turkey |
| Albert |  | Kaufmann | Cologne | Germany |
| Matthias |  | Katzmann | Bad Nauheim | Germany |
| Adham | Mohamad | Kassem | Beiruth | Lebanon |
| Simon |  | Kasa | Majuro | Marshall Islands |
| Kamila |  | Karmasova | Brno | Czech Republic |
| Ferhi |  | Karim | Rennes | France |
| Roman | Titus | Karig | Neuss | Germany |
| Neven |  | Kapun | Zagreb | Croatia |
| Rajni |  | Kapoor | Lucknow | India |
| Mohammad | Anas | Kanout | Coburg | Germany |
| Jacob |  | Kaneti | Beer-Sheva | Israel |
| Ktari | Ktari | Kamel | Monastir | Tunisia |
| Ahmed | Ismail | Kamel | Giza | Egypt |
| Odunayo |  | Kalejaiye | Plymouth | United Kingdom |
| Panagiotis |  | Kalafatis | Rhodos | Greece |
| Bela |  | Koves | Budapest | Hungary |
| Hikmet |  | Kãƒâ¶Seoã„Å¸Lu | IZMIR | Turkey |
| Mustafa |  | Kadihasanoglu | Istanbul | Turkey |
| Alina | No Name | Kaczmarek | Jozefoslaw | Poland |
| Brahim | Brahim | Kacem | Mostaganem | Algeria |
| Jan |  | Kaan | Utrecht | Netherlands |
| Suleyman |  | Karaca | Malatya | Turkey |
| Rozalia |  | Juraszik | Budapest | Hungary |
| Seung Il |  | Jung | Hwasun | Korea, South |
| Peter |  | Jung | Ellwangen | Germany |
| Noor | Nabi | Junejo | Karachi | Pakistan |
| Robert |  | Juhasz | Berettyoujfalu | Hungary |
| Kondas |  | Jozsef | Budapest | Hungary |
| Teemu | Juhani | Joutsi | Pori | Finland |
| Rafaãƒâ«L | M. | Joseph | Ieper | Belgium |
| Bruno | Alexandre Guerra | Jorge Pereira | Covilhãƒâ£ | Portugal |
| John | Jared | Johnson | Madrid | Spain |
| Antonio |  | Jimãƒâ©Nez-Pacheco | Granada | Spain |
| Ho | Jong | Jeon | Daegu | Korea, South |
| Susanne | Skou | Jensen | Aarhus N | Denmark |
| Ursula |  | Janzen | Hannover | Germany |
| Jiri |  | Janu | Nove Mesto Na Morave | Czech Republic |
| Vãƒâ¡Clav |  | Janda | Plzeã…Ë† | Czech Republic |
| Basharat | - | Jameel | Aberystwyth | United Kingdom |
| Lotte | Kaasgaard | Jakobsen | Aarhus | Denmark |
| Ramasamy |  | Jaganathan | Solihull | United Kingdom |
| Syed | Qasim | Jaffry | Galway | Ireland |
| Alessandro |  | Izzo | Napoli | Italy |
| Urii |  | Ivanov | Ekateringburg | Russia |
| George | Valerievich | Ivanov | Minsk | Belarus |
| Dmitri | D | Ivanov | Kiev | Ukraine |
| Untila | Victor | Iurie | Chisinev | Moldova |
| Rakasz |  | Istvan | Kaposvãƒâ¡R | Hungary |
| Vass |  | Istvãƒâ¡N | Miskolc | Hungary |
| Jamoliddin | Abdullajanov | Isroilovich | Namangan | Uzbekistan |
| Mohamed |  | Ismail | Bristol | United Kingdom |
| Noritaka |  | Ishito | Kurashiki | Japan |
| Satoshi |  | Ishihara | Minokamo, Gifu | Japan |
| Ekaterina | Martjushova | Irgens | Tromsãƒâ¸ | Norway |
| Jacques |  | Irani | Poitiers | France |
| Beniamino |  | Iorio | Roma | Italy |
| Hisato |  | Inatomi | Fukutsu | Japan |
| Hodi |  | Imre | Szolnok | Hungary |
| Ben Salah |  | Imed | Bizerte | Tunisia |
| Anthony | Chika | Igbokwe | Lagos | Nigeria |
| Alaa | Ahmed | Hussein | Cairo | Egypt |
| Akhtar Hussain |  | Hussain | Slough | United Kingdom |
| Emre |  | Huri | Ankara | Turkey |
| Tobias |  | Huebschle | Freiburg | Germany |
| Xiaoming |  | Huang | Shenyang | China |
| Andras |  | Hoznek | Creteil | France |
| Mustafa Burak |  | Hoşcan | Alanya-Antalya | Turkey |
| Titia | EM | Hopmans | Utrecht | Netherlands |
| Adel | Abdulkader | Hommos | Maadi, Cairo | Egypt |
| Petr |  | Holy | Praha | Czech Republic |
| Sten |  | Holmãng | Goteborg | Sweden |
| Endre |  | Holman | Kiskunhalas | Hungary |
| Reinhard | Hans Hermann | Hofmann | Wolfsburg | Germany |
| Harun |  | Hodzic | Zenica | Bosnia and Herzegovina |
| Andreas |  | Hinkel | Herne | Germany |
| Alexander | Ivanov | Hinev | Varna | Bulgaria |
| Mustafa |  | Hilmy | Stirling | United Kingdom |
| Vital |  | Hevia | Madrid | Spain |
| Jorge |  | Hernandez | Panama | Panama |
| Javier | Alejandro | Hernandez | Barquisimeto | Venezuela |
| David |  | Hernanadez | Santa Cruz De Tenerife | Spain |
| Jiri |  | Heracek | Prague | Czech Republic |
| Derek | B | Hennessey | Derry | United Kingdom |
| Flavio | L | Heldwein | Florianãƒâ³Polis | Brazil |
| Burkhard | Johannes | Held | Berlin | Germany |
| Mohamed Hani | Abdulwahab | Helal | Jeddah | Saudi Arabia |
| Ortwin |  | Heiãƒå¸Ler | Gansersdorf | Austria |
| Hiroshi |  | Hayami | Kagoshima | Japan |
| Simon | John | Hawkyard | Scarborough | United Kingdom |
| Botan |  | Hawas | Vasteras | Sweden |
| Gergely |  | Hausmann | Budapest | Hungary |
| Dinko |  | Hauptman | Zagreb | Croatia |
| Konstantinos |  | Hatzimouratidis | Pefka Thessaloniki | Greece |
| Ismail | M | Hassan | Dubai | United Arab Emirates |
| Hashim |  | Hashim | London | United Kingdom |
| Waleed | Ali | Hasan | Manama | Bahrain |
| Sergio | Pedro | Haro | Tromsoe | Norway |
| Ivan | Iovev | Handjiev | Varna | Bulgaria |
| Vishwanath | S | Hanchanale | York | United Kingdom |
| Chang | Hee | Han | Uijeongbu | Korea, South |
| Arman | H. | Hambaryan | Echmiadzin | Armenia |
| Ryoichi |  | Hamasuna | Kitakyushu | Japan |
| Ramin | -- | Hakimzadeh | Ormiyeh | Iran |
| Lars |  | Haggarth | Stockholm | Sweden |
| Susanne |  | Hagel | Berlin | Germany |
| Marios |  | Hadjipavlou | London | United Kingdom |
| Nassya | S. | Hadjieva | Sofia | Bulgaria |
| Perviz |  | Haciyev | Ankara | Turkey |
| Mohamad | Alsayed | Habous | Jedda | Saudi Arabia |
| U | Sin | Ha | Seoul | Korea, South |
| Prem | Raj | Gyawali | Kathmandu | Nepal |
| Laszlo |  | Gyanyi | Budapest | Hungary |
| Antonio |  | Gutierrez | L`Hospitalet | Spain |
| Necati |  | Gürbüz | Istanbul | Turkey |
| Cenk |  | Gurbuz | Istanbul | Turkey |
| Alfons |  | Gunnemann | Detmold | Germany |
| Adnan |  | Gündoğdu | İzmir | Turkey |
| Tawiz | Gul | Gul | Doha | Qatar |
| Serigne | Magueye | Gueye | Dakar | Senegal |
| Borut |  | Gubina | Ljubljana | Slovenia |
| Angelo |  | Guarriello | Andria | Italy |
| JesãƒâºS | Guajardo | Guajardo | Lleida | Spain |
| Gevorg | V. | Grigoryan | Yerevan | Armenia |
| Daniel |  | Grell | Berlin | Germany |
| Mireille |  | Gregoire | Quebec | Canada |
| Francesco |  | Greco | Halle Saale | Germany |
| H.J. |  | Graff | Solingen | Germany |
| Arthur |  | Grabsky | Yerevan | Armenia |
| Beata |  | Grabowska | Orebro | Sweden |
| Magnus | J | Grabe | Malmo | Sweden |
| Hesham | Saad | Gouda | Alexandria | Egypt |
| Eduardo |  | Gotuzzo | Lima | Peru |
| Dmitriy | Gennadievich | Goryainov | Donetsk | Ukraine |
| Sadik |  | Gorur | Antakya/Hatay | Turkey |
| Maximilien |  | Goris Gbenou | Valence | France |
| Mehmet Resit |  | Goren | Ankara | Turkey |
| Ravisankar |  | Gopakumarapillai | Manama | Bahrain |
| Juan | Uria | Gonzalez-Tova | Vic | Spain |
| Raquel |  | Gonzalez-Lopez | Madrid | Spain |
| Sergio | Norbert | Gonzalez-Bittner | Frankfurt | Germany |
| Francisco |  | Gomez Veiga | A Coruna | Spain |
| Yunus | Emre | Göğer | Konya | Turkey |
| Marcelo | Alvear | Godoy | Santiago | Chile |
| Alexander | Johannes | Glowik | Hannover | Germany |
| Bartek |  | Gliniewicz | Szczecin | Poland |
| Sidney |  | Glina | Sao Paulo | Brazil |
| Jochen |  | Gleissner | Wuppertal | Germany |
| Dimitrios |  | Glaritis | Heraklio | Greece |
| Canepa |  | Giorgio | Genoa | Italy |
| Fernando | J. | Giordano | Merida | Venezuela |
| Thomas |  | Gilbert | Koblenz | Germany |
| Achilles |  | Gikas | Heraklion | Greece |
| Vladimir |  | Giblo | Hradec Kralove | Czech Republic |
| Claudio | Antonio | Giberti | Chiavari | Italy |
| Artur |  | Gibas | Gdansk | Poland |
| Assem | Sayed-Ahmed | Ghallab | Tanta | Egypt |
| Sobhan |  | Ghafouryan | Ilam | Iran |
| Ardalan | Abdolghafouri | Ghafouri | Doha | Qatar |
| Ulrich |  | Gertenbach | Hagen | Germany |
| Lara |  | Gerbrandy-Schreuders | Amsterdam | Netherlands |
| Ventsislav | Dinkov | Georgiev | Ruse | Bulgaria |
| Georgios |  | Georgiadis | Redhill | United Kingdom |
| Andrey | Alexandravich | Gavrusev | Minsk | Belarus |
| Teresita | Tanaglin | Gaviola | Makati | Philippines |
| Sandro | Silva | Gaspar | Lisboa | Portugal |
| Ioannis | Dimitrios | Garifallos | Samos | Greece |
| Vanessa |  | Gardikou | Pireas | Greece |
| Francisco | Antonio | Garcia Velandria | Valencia | Venezuela |
| Antonio | Sãƒâ¡Nchez | Garcãƒâ­a | Linares | Spain |
| Maria |  | Garabasova | Trnava | Slovakia |
| Thirumalai |  | Ganesan | Chennai | India |
| Carlos |  | Gamiãƒâ±O | DF | Mexico |
| Catarina | Diogo | Gameiro | Lisboa | Portugal |
| Roman | Vladislavovich | Gamazkov | St-Petersburg | Russia |
| Xavier |  | Gamãƒâ© | Toulouse | France |
| David | J | Galvin | Dublin | Ireland |
| Michele |  | Gallucci | Rome | Italy |
| Natalya | Gennagievna | Galkina | Penza | Russia |
| Carlos | Pacheco | Gahbler | Mexico, D.F. | Mexico |
| Juan |  | Gãƒâ³Mez Rivas | Madrid | Spain |
| Sandor |  | Gecs | Veszprem | Hungary |
| Begench | Gurbangeldiyevich | Gadamov | Ashgabat | Turkmenistan |
| Dr Khaled | Abdul Moneim | Gadalla | Cairo | Egypt |
| Mauro |  | Gacci | Florence | Italy |
| Yaowen |  | Fu | Changchun | China |
| Hans-Martin |  | Fritsche | Regensburg | Germany |
| Cristina | Cristina | Freuler | Buenos Aires | Argentina |
| Olga |  | Fraschini | Lecco | Italy |
| Michail |  | Frank | Ekaterinburg | Russia |
| Duval |  | Francois | Reims | France |
| Gianfranco |  | Formicola | Napoli | Italy |
| Eleftherios | D | Fokaefs | Patras | Greece |
| Zoltãn |  | Florian | Nagykanizsa | Hungary |
| Christian |  | Fisang | Bonn | Germany |
| Juliane |  | Fiebich | Burgwedel | Germany |
| Mariaconsiglia |  | Ferriero | Rome | Italy |
| Vavassori | Ivano | Ferdinando | Bergamo | Italy |
| Pal |  | Fel | Dombovar | Hungary |
| Michal |  | Fedorko | Brno | Czech Republic |
| Gaiseniuk | Z | Fedir | Kiev | Ukraine |
| Narcisi |  | Federico | Tordino Teramo | Italy |
| Zsolt |  | Fazakas | Budapest | Hungary |
| Antal | - | Farkas | Debrecen | Hungary |
| Rui | Almeida | Farinha | Lisbon | Portugal |
| Yasser | Abd Elraouf | Farahat | Dubai | United Arab Emirates |
| Bernhard |  | Fangmeyer | Lingen | Germany |
| Walid |  | FALOU | Beirut | Lebanon |
| Siavash |  | Falahatkar | Rasht | Iran |
| Christian | Christian | Fahr | Reutlingen | Germany |
| Amogu | Kalu | Eziyi | Osogbo | Nigeria |
| Bakurov | E | Evgeny | Rostov-On-Don | Russia |
| Fleury |  | Estelle | Saint Aubin Sur Scie | France |
| Iouri | M | Essilevski | Moscow | Russia |
| Khadigeh |  | Esmaeili | Ilam | Iran |
| Erkan |  | Erkan | Istanbul | Turkey |
| Erbil |  | Ergenekon | Istanbul | Turkey |
| Seydali |  | Eredjepov | Ferghana | Uzbekistan |
| Erhan | - | Erdogan | Karaman | Turkey |
| Ibrahim |  | Erayman | Konya | Turkey |
| Carmen |  | Enguita | Madrid | Spain |
| Christina | Luise | Engels | Karlsruhe | Germany |
| Marcus |  | Elstermann Von Elster | Berlin | Germany |
| Emad | Rashad Mohamed | Elsobky | Abu Dhabi | United Arab Emirates |
| Alaa | Ali | Elshennawy | Cairo | Egypt |
| Medhat | Ahmed Mohamed | Elsayed | Abu Dhabi | United Arab Emirates |
| Maria | Carlsen | Elkjãƒâ¦R | Aarhus | Denmark |
| Rehab | Hosny | El-Sokkary | Zagazig | Egypt |
| Claus |  | Eisenreich | Bad Tolz | Germany |
| Solymossy |  | Egon | Székesfehérvár | Hungary |
| Christian |  | Eggersmann | Rheine | Germany |
| Ioannis | Panagiotis | Efthimiou | Chania | Greece |
| David |  | Ebralidze | Tbilisi | Georgia |
| Konstantin | Antonovich | Dunets | Saint-Petersburg | Russia |
| Athanasius | Daud | Dube | Harare | Zimbabwe |
| Kim | Maksut | Drasa | Tirana | Albania |
| Elisaveta | Asenova | Draghijeva | Kuwait | Kuwait |
| Tomasz |  | Drabarek | Gdansk | Poland |
| Elif |  | Doyuk Kartal | Eskisehir | Turkey |
| Roland |  | Donat | Edinburgh | United Kingdom |
| Andreas | Peter | Dominik | Giessen | Germany |
| Alexey | Alexeevitch | Dolgiy | Saint-Petersburg | Russia |
| Razvan | Vasile | Dican | Hof | Germany |
| Diab | El-Sayed | Diab | Zagazig | Egypt |
| Manuel |  | Di Biase | Perugia | Italy |
| Laura | Frances | Derbyshire | Salford | United Kingdom |
| Nese |  | Demirturk | Afyon | Turkey |
| Erhan |  | Demirelli | Giresun | Turkey |
| Umut |  | Delibas | Istanbul | Turkey |
| Giulio |  | Del Popolo | Firenze | Italy |
| Kojic | Dusan | Dejan | Belgrade | Serbia and Montenegro |
| Liana | Monica | Deac | Cluj-Napoca | Romania |
| Geert |  | De Naeyer | Aalst | Belgium |
| Jose | Carneiro | De Moura | Lisbon | Portugal |
| Ferdinando |  | De Marco | Grottaferrata (Rome) | Italy |
| Siviardo | Cristobal | De Leon | Zapopan Jalisco | Mexico |
| Gaetano |  | De Grande | Siracusa | Italy |
| Gioacchino |  | De Giorgi | Udine | Italy |
| Domenico |  | De Carolis | Ascoli P | Italy |
| Mark |  | Davis | Newcastle Upon Tyne | United Kingdom |
| John | Brinley | Davies | Newcastle Upon Tyne | United Kingdom |
| Anton |  | Dashko | Moscow | Russia |
| Amitabh |  | Dash | Delhi | India |
| Debi | Narayan | Das Chaudhury | PERTH | United Kingdom |
| Fontana |  | Dario | Torino | Italy |
| Ziad |  | Daoud | Tripoli | Lebanon |
| Quang Oanh |  | Dao | Hochiminh | Vietnam |
| Michael |  | Dan | Holon | Israel |
| Le Dinh |  | Dam | Les Abymes | Guadeloupe |
| Izak |  | Dalva | Ankara | Turkey |
| Hani | R | Dahmash | Der Ezore | Syria |
| Hugo | Alberto | Dãƒâ¡Vila | Caracas | Venezuela |
| Xhevdet | M. | Cuni | Prishtina | Albania |
| Bogdan | A. | Culibrk | Subotica | Serbia and Montenegro |
| Jose | Angel | Cuesta-Alcalãƒâ¡ | Tudela (Navarra) | Spain |
| Judit |  | Csorba | Debrecen | Hungary |
| Cristiano |  | Cristini | Rome | Italy |
| Pietro |  | Cozzupoli | Reggio Calabria | Italy |
| Pedro |  | Coteron | Murcia | Spain |
| Elisabetta |  | Costantini | Perugia | Italy |
| Bogdan |  | Costache | ARAD | Romania |
| Tiago | Pinto | Correia | Matosinhos | Portugal |
| Nick |  | Cooley | London | United Kingdom |
| Sophie |  | Conquy | Paris | France |
| Nicholas |  | Cohen | Aberdeen | United Kingdom |
| Manuel | Ferreira | Coelho | Amadora | Portugal |
| Andrea |  | Cocci | Firenze | Italy |
| Liliana | Ofelia | Clara | Buenos Aires | Argentina |
| Matteo | R. | Ciuffreda | Parma | Italy |
| Sinharib |  | Citgez | Istanbul | Turkey |
| Ettore |  | Cirillo Marucco | Andria | Italy |
| Janusz | Andrzej | Ciechan | Lublin | Poland |
| Izzet |  | Cicekbilek | Zonguldak | Turkey |
| Jae Min |  | Chung | Busan | Korea, South |
| Hong |  | Chung | Chungju | Korea, South |
| Susanne |  | Christmann | Ludwigshafen | Germany |
| Michael |  | Chrisofos | Athens | Greece |
| Liaqat |  | Chowoo | Scunthorpe | United Kingdom |
| Kostas | Nik | Chondros | Heraklion | Greece |
| Jin-Bong | . | Choi | Gyeonggi-Do | Korea, South |
| Hyun-Sop |  | Choe | Suwon | Korea, South |
| Yong-Hyun |  | Cho | Seoul | Korea, South |
| Wonyeol |  | Cho | Busan | Korea, South |
| Kang Jun |  | Cho | Bucheon-City | Korea, South |
| In-Rae |  | Cho | Koyang/Gyunggido | Korea, South |
| Archil |  | Chkhotua | Tbilisi | Georgia |
| Daniel | Mihai | Chirita | Piatra Neamt | Romania |
| Edmund |  | Chiong | Singapore | Singapore |
| Xi |  | Cheng | Taunton | United Kingdom |
| Yee-Chun |  | Chen | Taipei | Taiwan |
| Ming |  | Chen | Nanjing | China |
| Christophe |  | Chemaslãƒâ€° | Palmerston North | New Zealand |
| Stavros | N | Charalambous | THESSALONIKI | Greece |
| Sammy | K K | Chan | Hong Kong | Hong Kong |
| Suchart |  | Chaimuangraj | Bangkok | Thailand |
| Mehmet |  | Cetinkaya | Istanbul | Turkey |
| Mete |  | Çek | Edirne | Turkey |
| Treuthardt |  | Cedric | Lausanne | Switzerland |
| Diego | Martin | Cecchini | Buenos Aires | Argentina |
| Oğuz | Özden | Cebeci | Ankara | Turkey |
| Nelson | A. | Cayco | Cabanatuan | Philippines |
| Luis | Augusto | Castro Sãƒâ¡Der | Barcelona | Spain |
| Octavio | Augusto | Castillo | Santiago | Chile |
| Daniele | D | Castellani | Viterbo | Italy |
| Turhan |  | Caskurlu | Istanbul | Turkey |
| Biljana | Luka | Carevic | Belgrade | Serbia and Montenegro |
| Andrãƒâ© | Martins | Cardoso | Matosinhos | Portugal |
| Enrique |  | Cao Avellaneda | Murcia (Murcia) | Spain |
| Yanwei |  | Cao | Qingdao | China |
| Zeynel |  | Canoğulları | Turkey | Turkey |
| Josep |  | Campa | Gasteiz | Spain |
| Chibelean | Bogdan | Calin | Bucharest | Romania |
| Emanuele |  | Caldarera | Palermo | Italy |
| Tiziana |  | Calcagno | Genova | Italy |
| Adriano | Almeida | Calado | Recife | Brazil |
| Basri |  | Çakıroğlu | Istanbul | Turkey |
| Tommaso |  | Cai | Trento | Italy |
| Josa | Pedro | Cadilhe | Viana Castelo | Portugal |
| Susana | Gabriela | Cabrera | Montevideo | Uruguay |
| Juan | Pablo | Caballero | Alicante | Spain |
| Xhemil | Ramiz | Bytyci | Prishtina-Kosovo | Serbia and Montenegro |
| Istvãn |  | Buzogãny | Budapest | Hungary |
| Joerg |  | Busche | Diepholz | Germany |
| Almudena |  | Burillo | Madrid | Spain |
| Stefan |  | Buntrock | Arendal | Norway |
| Burak | Besir | Bulut | Kahramanmaras | Turkey |
| Hubert |  | BUGEL | ELBEUF | France |
| Igor |  | Brzakovic | Novi Sad | Serbia and Montenegro |
| Franck |  | Bruyere | Tours | France |
| Sebastiano |  | Bruschetta | Messina | Italy |
| Milos |  | Brodak | Hradec Kalove | Czech Republic |
| Oliver |  | Brock | Hof | Germany |
| Elena | Vladimirovna | Brizhatyuk | Novosibirsk | Russia |
| David |  | Brix | Wuerzburg | Germany |
| Konstantinos |  | Bratsas | Haidari, Athens | Greece |
| Frederico | A.D. | Branco | Porto | Portugal |
| Tommaso |  | Brancato | Albano Laziale | Italy |
| Ugur |  | Boylu | Istanbul | Turkey |
| Pierluigi |  | Bove | Rome | Italy |
| Philip | S | Bova | Rostov-On-Don | Russia |
| Andreas |  | Bourdoumis | Exeter | United Kingdom |
| Amine |  | Bouassida | Lens | France |
| Henry |  | Botto | Suresnes | France |
| Nagy |  | Botond | Brasov | Romania |
| Francisco | J. Santos | Botelho | Porto | Portugal |
| Pia | Carla | Bossola | Domodossola | Italy |
| Ricardo | G. | Borges | Coimbra | Portugal |
| Gernot |  | Bonkat | Basel | Switzerland |
| Giovanni |  | Bonfiglio | Catania | Italy |
| Ahmet |  | Bolukbasi | Izmir | Turkey |
| Jean-Paul |  | Boiteux | Clermont-Ferrand | France |
| Sotirios |  | Bogris | London | United Kingdom |
| Csaba |  | Bognãƒâ¡R | Miskolc | Hungary |
| Malte |  | Boehm | Dillenburg | Germany |
| Katharina |  | Boehm | Hamburg | Germany |
| Balazs |  | Bodrogi | Miskolc | Hungary |
| Franco |  | Blefari | Orvieto | Italy |
| Brian | Penero | Blas | Tacloban, Leyte | Philippines |
| Truls | Erik | Bjerklund Johansen | Oslo | Norway |
| Jure |  | Bizjak | Ljubljana | Slovenia |
| Werner | H | Bischoff | Backnang | Germany |
| Rasmus |  | Bisbjerg | Herlev | Denmark |
| Brian | Robert | Birch | Southampton | United Kingdom |
| Renu |  | Bharadwaj | Pune | India |
| Aashutosh |  | Bhanot | Pleven | Bulgaria |
| Shashikant | R | Bhange | Uttam Plaza, Pune | India |
| Burkhard |  | Beyer | Hamburg | Germany |
| Ingrid | H | Berger | Wien | Austria |
| Boris | Vitalyevitch | Berejanski | Moscow | Russia |
| Eduardo |  | Bercowsky | Menorca | Spain |
| Saida |  | Benredjeb | Tunis | Tunisia |
| Francesco |  | Beniamin | Treviso | Italy |
| Giuseppe |  | Benedetto | Padova | Italy |
| Nawfel |  | Ben Rais | Tunis | Tunisia |
| Payam |  | BEHZADI | Tehran | Iran |
| Selahattin |  | Bedir | Ankara | Turkey |
| Jaroslav |  | Beck | Kosice | Slovakia |
| Pastora |  | Beardo | Jerez De La Frontera | Spain |
| Mustafa |  | Bazardzanovic | Tuzla | Bosnia and Herzegovina |
| Rıza |  | Baysal | Antalya | Turkey |
| Yildirim |  | Bayazit | Adana | Turkey |
| Mihir | V | Baxi | Mumbai | India |
| Michael |  | Baumann | Hameln | Germany |
| Stojan |  | Batandjiovski | Bitola | Macedonia, The Former Yugoslav Republic of |
| Paulo | Roberto | Bastos | Niteroi Rio De Janeiro | Brazil |
| Ralf | F. | Basting | Altoetting | Germany |
| Harry |  | Bassaris | Patras | Greece |
| Gediminas |  | Baseckas | Ystad | Sweden |
| Riccardo |  | Bartoletti | Pistoia | Italy |
| Joeri |  | Barth | Ghent | Belgium |
| Lyidmila | Pavlovna | Barashova | Kemerovo | Russia |
| Caner |  | BARAN | Kahramanmaras | Turkey |
| Firuz |  | Barakaev | Moscow | Russia |
| Maria | Del Carmen | Bangher | Corrientes | Argentina |
| Vladimir | Mihailo | Bancevic | Belgrade | Serbia and Montenegro |
| Goran | Krume | Balevski | Ohrid | Macedonia, The Former Yugoslav Republic of |
| Melih |  | Balci | Ankara | Turkey |
| Mehmet |  | Balasar | Konya | Turkey |
| Mohammad |  | Bakhtiar | London | United Kingdom |
| Senad |  | Bajramovic | Sarajevo | Bosnia and Herzegovina |
| Gabor |  | Bajor | Budapest | Hungary |
| Seung |  | Baik | Gwang-Ju | Korea, South |
| Badrulhisham |  | Bahadzor | Cheras, KL | Malaysia |
| Mohamed | Alaa | Bahaa | Nyala | Sudan |
| Mahmood | Reza | Baghinia | Arak | Iran |
| Marcos | Alessandro | Baeza | Umuarama Pr | Brazil |
| Jae Hyun |  | Bae | Ansan | Korea, South |
| Cãƒâ©Cile |  | BACH | Suresnes | France |
| Birgit | Kaa | Bach | Aarhus N | Denmark |
| Abdul | Azim | Azizi | Herlev | Denmark |
| Olgun |  | Azis | Constanta | Romania |
| Behrooz | Rahnavardi | Azari | Bandar Abbas | Iran |
| Omer |  | Aytac | Istanbul | Turkey |
| Serdar |  | Aykan | Istanbul | Turkey |
| Elin |  | Axen | Gothenburg | Sweden |
| M Hammad |  | Ather | Karachi | Pakistan |
| Grigorios |  | Athanasiadis | Guildford | United Kingdom |
| Muhammad |  | ATFA | Aulnay Sous Bois | France |
| Ferhat |  | Ates | Istanbul | Turkey |
| Henry | None | Atawurah | Takoradi | Ghana |
| Rasul | Akhmedjanovich | Ataniyazov | Tashknet | Uzbekistan |
| Ali |  | Atan | Ankara | Turkey |
| Mehmet | Kazim | Asutay | Istanbul | Turkey |
| Mohamadali |  | Aslmonadi | Tabriz | Iran |
| Yilmaz |  | Aslan | Ankara | Turkey |
| Ergul |  | Aslan | Istanbul | Turkey |
| Ahmet | Ruknettin | Aslan | Istanbul | Turkey |
| Ramazan |  | Asci | Samsun | Turkey |
| Cagri |  | Asan | BATMAN | Turkey |
| Montserrat |  | Arzoz | Badalona | Spain |
| Levon | Dm | Arustamov | Tashkent | Uzbekistan |
| Burak |  | Arslan | Istanbul | Turkey |
| Nasr |  | Arsanious | Croydon | United Kingdom |
| Szanto |  | Arpad | Pecs | Hungary |
| Athanasios | N | Argyropoulos | Elefsina | Greece |
| Manuel | Alvarez | Ardura | Alcorcon, Madrid | Spain |
| Ersan |  | Arda | Edirne | Turkey |
| Alejandro |  | Arce | Mexico | Mexico |
| Saeid |  | Arasteh | Bam | Iran |
| Haykaz |  | Antonyan | Yerevan | Armenia |
| Abi Aad |  | Antoine | Brussels | Belgium |
| Javier |  | Angulo | Getafe, Madrid | Spain |
| Catarina | Ingrid | Aneman | Goteborg | Sweden |
| Alexander | Vladimirovich | Andreychikov | Krasnoyarsk | Russia |
| Elena |  | Andretta | Dolo (Venice) | Italy |
| Kim | Hovgaard | Andreassen | Fredericia | Denmark |
| Anders | Christian | Andersson | Limhamn | Sweden |
| Leif | Percival | Andersen | Copenhagen Ãƒëœ | Denmark |
| Jens | Thorup | Andersen | Roskilde | Denmark |
| Polvonov | Abror | Aminovich | Samarkand | Uzbekistan |
| Miguel |  | Alvarez-Mugica | Oviedo | Spain |
| S B Pavan Kumar |  | Aluru | Sunderland | United Kingdom |
| Muammer |  | Altok | Izmir | Turkey |
| Mustafa |  | Altindis | Sakarya | Turkey |
| Javier | David | Altclas | Buenos Aires | Argentina |
| Nayel | Abdullah | Altarawneh | Doha | Qatar |
| Berthold |  | Alt | Fulda | Germany |
| Naimet | Kamal | Alsaigh | Alain - Abudhabi | United Arab Emirates |
| Abdulmunem | Mohammed | Alsadi | Dubai | United Arab Emirates |
| Caner |  | Alptekin | Izmir | Turkey |
| Ahmed | Mabrouk | Alosta | London | United Kingdom |
| Vafa | Abd | Allahpour | Mahabad | Iran |
| Ahmad | Ashhab | Alkadi | Ryiad | Saudi Arabia |
| Ahmed | Khalil | Aljubory | Mosul | Iraq |
| Jakhongir | Fatikhovich | Alidjanov | Tashkent | Uzbekistan |
| Tarek |  | Ali | Nyiregyhaza | Hungary |
| Arif | Maqsood | Ali | Okara | Pakistan |
| Ahmed | Ragab | Ali | Sharkia | Egypt |
| Ammar |  | Alhasso | Dunfermline | United Kingdom |
| Gamal | Mohammed Sami | Alhadad | Alexandria | Egypt |
| Naser | Abdussalam | Alfgi | Tripoli | Libya |
| Shulyak |  | Alexander | Lviv | Ukraine |
| Egote | Kofi | Alexander | Moscow | Russia |
| Dmitry |  | Aleshin | Brno | Czech Republic |
| Mohammad | Khalil Ibrahim | Aldahiri | Abu Dhabi | United Arab Emirates |
| Emilio | Lopez | Alcina | Valencia | Spain |
| Istvan |  | Albert | Budapest | Hungary |
| Zaheer |  | Alam | Karachi | Pakistan |
| Bulent |  | ALAGOL | Edirne | Turkey |
| Muna | Mohsen | Al-Haidary | Hodeidah | Yemen |
| Kais |  | Al-Dairi | Chelmsford | United Kingdom |
| Nahed | Ahmad | Al Tabash | Taif | Saudi Arabia |
| Riyadh |  | Al Salh | Dammam | Saudi Arabia |
| Ilker | - | Akyol | Istanbul | Turkey |
| Sergey | Dmitrievich | Aksionov | Kiev | Ukraine |
| Yigit |  | Akin | Antalya | Turkey |
| Waseem |  | Akhter | London | United Kingdom |
| Hacã„Â± | Murat | Akgãƒâ¼L | Istanbul | Turkey |
| Ali | Ferruh | Akay | Istnbul | Turkey |
| Ulrike |  | Ahrens | Ulm | Germany |
| Jae Hyun |  | Ahn | Yangsan-Si, Gyeongnam | Korea, South |
| Mumtaz |  | Ahmad | Rawalpindi | Pakistan |
| Christer |  | Ahlstrand | Linköping | Sweden |
| Reza |  | Aghelnezhad | Mashhad | Iran |
| Mehmet |  | Oã„Å¸Lu | Gaziantep | Turkey |
| Akisibadek | Alekz | Afoko | Tamale | Ghana |
| Vasileios | Georgios | Adamopoulos | Heraklio | Greece |
| Przemyslaw |  | Adamczyk | Torun | Poland |
| Iwan | Asmara | Achmad | Jakarta | Indonesia |
| Kamal | Jamil | Achkar | Creil | France |
| Ömer |  | Acar | Istanbul | Turkey |
| Refaat | Mohamad | Abusamra | Misrata | Libya |
| Tarik | Sulaiman | Abulhul | Tripoli | Libya |
| Salisu |  | Abubakar | Kano | Nigeria |
| Leif |  | Abramsson | Umeãƒâ¥ | Sweden |
| Ernesto | A | Aboytes | Celaya | Mexico |
| Vitaly | Eduardovich | Aboyan | Rostov-On-Don | Russia |
| Mohamed |  | Abo El-Enen | Tanta | Egypt |
| Mohamed | Ismat | Abdulmajed | Milton Keynes | United Kingdom |
| Iskander | Ilfakovich | Abdullin | Moscow | Russia |
| Muthanna | Saad | Abduljawad | Mosul | Iraq |
| Bouzouita |  | Abderrazak | Tunis | Tunisia |
| Amr | Mahmoud | Abdelhakim | Cairo | Egypt |
| Khaled | Farid | Abdel-Aziz | Dallas | United States |
| Hatem | Mahrous | Abdel Wahab | Cairo | Egypt |
| Antonino |  | Abbolito | Roma | Italy |
| Bruno |  | Abbate | Florence | Italy |
| Malashchitsky | Dmitry | A. | Minsk | Belarus |
