# Supplementary material for: Appropriate empiric antibiotic choices in health care associated urinary tract infections in urology departments in Europe from 2006 to 2015: A Bayesian analytical approach applied in a surveillance study
Source: PLoS One. 2019 Apr 25;14(4):e0214710. doi: 10.1371/journal.pone.0214710 (PMC6483335; doi:10.1371/journal.pone.0214710)
Supplement: S5 Appendix — (DOCX) [file pone.0214710.s007.docx]

# **S5 Appendix. References used in supporting information sections.**

1. David J. Spegelhalter, K.R.A., Jonathan P. Myles. *Bayesian Approaches to Clinical Trials and Health-Care Evaluation*, (2003).

2. Huber, W.*, et al.* Orchestrating high-throughput genomic analysis with Bioconductor. *Nature methods* **12**, 115-121 (2015).

3. Briggs, A.H., Ades, A.E. & Price, M.J. Probabilistic sensitivity analysis for decision trees with multiple branches: use of the Dirichlet distribution in a Bayesian framework. *Medical decision making : an international journal of the Society for Medical Decision Making* **23**, 341-350 (2003).

4. Leclercq, R.*, et al.* EUCAST expert rules in antimicrobial susceptibility testing. *Clinical microbiology and infection : the official publication of the European Society of Clinical Microbiology and Infectious Diseases* **19**, 141-160 (2013).

5. Briggs, A., Sculpher, M. & Claxton, K. *Decision modelling for health economic evaluation*, (OUP Oxford, 2006).
