# Supplementary material for: Appropriate empiric antibiotic choices in health care associated urinary tract infections in urology departments in Europe from 2006 to 2015: A Bayesian analytical approach applied in a surveillance study
Source: PLoS One. 2019 Apr 25;14(4):e0214710. doi: 10.1371/journal.pone.0214710 (PMC6483335; doi:10.1371/journal.pone.0214710)
Supplement: S1 Table — (DOCX) [file pone.0214710.s008.docx]

# S1 Table. Antibiotic choices evaluated for empirical treatment of HAUTIs in urology.

| Single antibiotic choices | Combination antibiotics |
| --- | --- |
| Aminopenicillin/Beta lactamase inhibitor (Amp/BLI) | Amp/BLI+Gentamicin |
| Piperacillin/Tazobactam(TZB) | Ciprofloxacin+ Gentamicin |
| Cefuroxime | Piperacillin/Tzb + Gentamicin |
| Cefepime | Ceftazidime+ Gentamicin |
| Cefotaxime | Imipenem+ Gentamicin |
| Ceftazidime | Ciprofloxacin+ Amikacin |
| Ciprofloxacin | Ceftazidime+Amikacin |
| Gentamicin | Imipenem+Amikacin |
| Amikacin |  |
| Imipenem |  |
