# Supplementary material for: Appropriate empiric antibiotic choices in health care associated urinary tract infections in urology departments in Europe from 2006 to 2015: A Bayesian analytical approach applied in a surveillance study
Source: PLoS One. 2019 Apr 25;14(4):e0214710. doi: 10.1371/journal.pone.0214710 (PMC6483335; doi:10.1371/journal.pone.0214710)
Supplement: S2 Table — A. Number of departments from each European country per year. (“x” refers to no registries from the country on the GPIU study year). B. Number of patients surveyed and the prevalence of HAUTIS per year. (DOCX) [file pone.0214710.s009.docx]

**S2 Table A. Number of departments from each European country per year.** (“x” refers to no registries from the country on the GPIU study year).

|  | 2005 | 2006 | 2007 | 2008 | 2009 | 2010 | 2011 | 2012 | 2013 | 2014 | 2015 |
| --- | --- | --- | --- | --- | --- | --- | --- | --- | --- | --- | --- |
| Albania | x | x | x | x | x | x | x | x | x | 1 | x |
| Austria | 3 | x | 1 | x | x | 1 | 1 | x | x | 1 | x |
| Belgium | x | 1 | x | x | x | x | x | x | x | x | 1 |
| Bosnia and Herzegovina | 2 | 1 | 1 | x | 2 | 2 | 1 | 1 | 1 | 1 | 1 |
| Bulgaria | 2 | 1 | 1 | x | x | x | x | x | x | x | 1 |
| Croatia | x | x | x | 1 | x | x | x | x | 1 | x | 1 |
| Czech Republic | 3 | 1 | 1 | 1 | 2 | 2 | 2 | x | x | 3 | x |
| Denmark | x | x | 2 | 1 | 1 | 3 | 1 | 1 | 1 | x | 1 |
| Estonia | x | x | x | x | x | x | x | x | x | 1 | 1 |
| Germany | 37 | 10 | x | 10 | 5 | 9 | 6 | x | 16 | 8 | 2 |
| Finland | 1 | x | x | x | x | x | x | x | x | x | x |
| France | 4 | x | 1 | x | x | x | x | x | x | x | x |
| Greece | 2 | 6 | 4 | 5 | 2 | 4 | 3 | x | 1 | 2 | 1 |
| Hungary | 21 | 9 | 12 | 9 | 10 | 12 | 7 | x | 2 | 3 | 2 |
| Italy | 4 | 4 | 5 | 4 | 3 | 7 | 6 | 1 | 7 | 7 | 1 |
| Ireland | x | x | x | x | x | x | x | x | x | 1 | 1 |
| FYROM | 1 | x | x | x | x | 1 | 2 | x | x | 1 | x |
| Netherlands | 1 | x | x | x | x | x | 1 | x | 1 | 1 | x |
| Norway | 3 | x | x | x | x | x | x | x | 1 | 1 | 2 |
| Poland | x | x | 1 | x | 1 | 2 | 1 | x | x | 1 | 2 |
| Portugal | 1 | x | x | 2 | 1 | 4 | 2 | 1 | 6 | 5 | 2 |
| Romania | 1 | x | x | 1 | x | x | 2 | x | 1 | 1 | 1 |
| Serbia | x | x | x | x | x | x | x | x | x | 3 | x |
| Slovakia | 1 | 1 | 1 | 1 | 1 | 2 | 2 | x | 1 | 1 | x |
| Spain | 8 | 1 | 2 | 1 | 4 | 5 | 7 | x | 3 | 7 | 4 |
| Sweden | 1 | 5 | 2 | 3 | 1 | 2 | 2 | 1 | 2 | 1 | x |
| Switzerland | 2 | x | 1 | 1 | 1 | 2 | 1 | x | x | 1 | x |
| Turkey | 16 | 9 | 8 | 11 | 2 | 7 | 5 | x | 3 | 7 | 3 |
| UK | x | 1 | 7 | 2 | 3 | 10 | 9 | x | 6 | 8 | 3 |
| Total Number of Countries from Europe | 20 | 13 | 16 | 15 | 15 | 17 | 19 | 5 | 16 | 23 | 18 |

**S2 Table B. Number of patients surveyed and the prevalence of HAUTIS per year.**

|  | 2005 | 2006 | 2007 | 2008 | 2009 | 2010 | 2011 | 2012 | 2013 | 2014 | 2015 |
| --- | --- | --- | --- | --- | --- | --- | --- | --- | --- | --- | --- |
| Number of patients surveyed from Europe | 2,643 | 1,458 | 1,731 | 1,440 | 1,008 | 1,946 | 1,538 | 1,026 | 1,392 | 1,325 | 353 |
| Prevalence of HAUTs with microbiological proof of infection excluding ABU and MAGI | 3.9% | 3.8% | 5.5% | 6.9% | 5.2% | 4.4% | 11.8% | 4.8% | 10.8% | 7.9% | 12.5% |
