# Supplementary material for: Appropriate empiric antibiotic choices in health care associated urinary tract infections in urology departments in Europe from 2006 to 2015: A Bayesian analytical approach applied in a surveillance study
Source: PLoS One. 2019 Apr 25;14(4):e0214710. doi: 10.1371/journal.pone.0214710 (PMC6483335; doi:10.1371/journal.pone.0214710)
Supplement: S3 Table — (DOCX) [file pone.0214710.s010.docx]

# **S3 Table. Pathogens identified in the annual surveillance study of GPIU in Europe.**

|  | 2005 | | 2006 | | 2007 | | 2008 | | 2009 | | 2010 | | 2011 | | 2012 | | 2013 | | | 2014 | | | 2015 | |
| --- | --- | --- | --- | --- | --- | --- | --- | --- | --- | --- | --- | --- | --- | --- | --- | --- | --- | --- | --- | --- | --- | --- | --- | --- |
|  | n | % | n | % | n | % | n | % | n | % | n | % | n | % | n | % | n | % | n | | % | n | | % |
| Acinetobacter | 1 | 0.88 | 1 | 1.67 | 2 | 1.82 | 1 | 0.98 | 1 | 1.52 | 0 | 0 | 1 | 0.49 | 0 | 0 | 1 | 0.63 | 3 | | 1.81 | 0 | | 0 |
| Citrobacter | 2 | 1.77 | 2 | 3.33 | 0 | 0 | 2 | 1.96 | 0 | 0. | 2 | 2.06 | 3 | 1.46 | 0 | 0 | 0 | 0 | 1 | | 0.60 | 0 | | 0 |
| CoNS | 3 | 2.65 | 1 | 1.67 | 2 | 1.82 | 0 | 0 | 1 | 1.52 | 2 | 2.06 | 5 | 2.43 | 0 | 0 | 2 | 1.27 | 1 | | 0.60 | 0 | | 0 |
| E.coli | 46 | 40.71 | 29 | 48.33 | 43 | 39.09 | 44 | 43.14 | 31 | 46.97 | 40 | 41.24 | 92 | 44.66 | 33 | 78.57 | 76 | 48.10 | 76 | | 45.78 | 14 | | 25.93 |
| Enterobacter | 0 | 0.00 | 5 | 8.33 | 9 | 8.18 | 5 | 4.90 | 3 | 4.55 | 2 | 2.06 | 9 | 4.37 | 0 | 0.00 | 9 | 5.70 | 8 | | 4.82 | 4 | | 7.41 |
| Enterococcus sp. | 14 | 12.39 | 6 | 10 | 9 | 8.18 | 8 | 7.84 | 8 | 12.12 | 12 | 12.37 | 28 | 13.59 | 4 | 9.52 | 11 | 6.96 | 19 | | 11.45 | 6 | | 11.11 |
| Klebsiella sp. | 15 | 13.27 | 7 | 11.67 | 15 | 13.64 | 12 | 11.76 | 5 | 7.58 | 12 | 12.37 | 19 | 9.22 | 2 | 4.76 | 16 | 10.13 | 21 | | 12.65 | 8 | | 14.81 |
| Morganella | 0 | 0 | 0 | 0 | 0 | 0 | 0 | 0 | 0 | 0 | 0 | 0 | 1 | 0.49 | 0 | 0 | 0 | 0 | 0 | | 0 | 0 | | 0 |
| Other bacteria | 2 | 1.77 | 0 | 0 | 5 | 4.55 | 0 | 0 | 2 | 3.03 | 3 | 3.09 | 5 | 2.43 | 0 | 0 | 5 | 3.16 | 8 | | 4.82 | 4 | | 7.41 |
| Other Enterobacteriacea | 2 | 1.77 | 0 | 00 | 3 | 2.73 | 2 | 1.96 | 2 | 3.03 | 3 | 3.09 | 5 | 2.43 | 0 | 0 | 2 | 1.27 | 1 | | 0.6 | 3 | | 5.56 |
| Other Gr(+) | 1 | 0.88 | 1 | 1.67 | 2 | 1.82 | 1 | 0.98 | 3 | 4.55 | 1 | 1.03 | 2 | 0.97 | 0 | 0 | 6 | 3.80 | 4 | | 2.41 | 1 | | 1.85 |
| Other non-fermenter | 1 | 0.88 | 0 | 0 | 0 | 0 | 0 | 0 | 0 | 0 | 0 | 0 | 0 | 0 | 0 | 0 | 1 | 0.63 | 0 | | 0 | 1 | | 1.85 |
| Proteus sp. | 8 | 7.08 | 1 | 1.67 | 10 | 9.09 | 8 | 7.84 | 1 | 1.52 | 5 | 5.15 | 10 | 4.85 | 0 | 0 | 9 | 5.70 | 10 | | 6.02 | 3 | | 5.56 |
| P.aeruginosa | 10 | 8.85 | 3 | 5 | 5 | 4.55 | 17 | 16.67 | 5 | 7.58 | 12 | 12.37 | 17 | 8.25 | 3 | 7.14 | 8 | 5.06 | 11 | | 6.63 | 7 | | 12.96 |
| S.aureus | 5 | 4.42 | 2 | 3.33 | 4 | 3.64 | 1 | 0.98 | 2 | 3.03 | 3 | 3.09 | 6 | 2.91 | 0 | 0.00 | 5 | 3.16 | 1 | | 0.60 | 1 | | 1.85 |
| Candida albicans | 2 | 1.77 | 2 | 3.33 | 1 | 0.91 | 1 | 0.98 | 2 | 3.03 | 0 | 0 | 3 | 1.46 | 0 | 0 | 7 | 4.43 | 2 | | 1.20 | 2 | | 3.70 |
| Other fungi | 1 | 0.88 | 0 | 0 | 0 | 0 | 0 | 0 | 0 | 0 | 0 | 0 | 0 | 0 | 0 | 0 | 0 | 0 | 0 | | 0 | 0 | | 0 |
| total | 113 | 100 | 60 | 100 | 110 | 100 | 102 | 100 | 66 | 100 | 97 | 100 | 206 | 100 | 42 | 100 | 158 | 100 | 166 | | 100 | 54 | | 100 |
