# Supplementary material for: Appropriate empiric antibiotic choices in health care associated urinary tract infections in urology departments in Europe from 2006 to 2015: A Bayesian analytical approach applied in a surveillance study
Source: PLoS One. 2019 Apr 25;14(4):e0214710. doi: 10.1371/journal.pone.0214710 (PMC6483335; doi:10.1371/journal.pone.0214710)
Supplement: S4 Table — (DOCX) [file pone.0214710.s011.docx]

**S4 Table. Number of patients in departments with different infection control practices.**

|  | 2006 | 2007 | 2008 | 2009 | 2010 | 2011 | 2012 | 2013 | 2014 | 2015 |
| --- | --- | --- | --- | --- | --- | --- | --- | --- | --- | --- |
| Complete adherence | (n:43) | (n:73) | (n:70) | (n:44) | (n:54) | (n:84) | (n:11) | (n:111) | (n:92) | (n:33) |
| Partial adherence | (n:9) | (n:17) | (n:14) | (n:11) | (n:22) | (n29:) | (n:6) | (n:26) | (n:26) | (n:2) |
| Absence of adherence | (n:7) | (n:18) | (n:18) | (n:11) | (n:21) | (n:31) | (n:3) | (n:10) | (n:4) | (n:8) |
